# Meta-Analyzing the Impacts of Social Robots on Children’s Social and Emotional Development over Two Decades

**DOI:** 10.3390/jintelligence14100246

**Published:** 2026-10-04

**Authors:** Kejun Zhang, Zaipeng Zhang, Wenjia Cui, Zhen Gao, Cixian Lv, Taghreed Ali Alsudais, Xinghua Wang

**Affiliations:** 1 School of Education Science, Qingdao University, Qingdao 266071, China; zhangkejun1@qdu.edu.cn (K.Z.); zhangzaipeng@qdu.edu.cn (Z.Z.); 2 Moray House School of Education and Sport, The University of Edinburgh, Old College, South Bridge, Edinburgh EH8 9YL, UK; s2848738@ed.ac.uk; 3 School of Education and Human Development, The Education University of Hong Kong, Hong Kong, China; s1162290@s.eduhk.hk; 4 College of Education and Human Development, Princess Nourah bint Abdulrahman University, Riyadh 11671, Saudi Arabia; taalsudais@pnu.edu.sa; 5 Faculty of Applied Sciences, Macao Polytechnic University, Macao 999078, China

**Keywords:** social robot, social emotional learning, children development, meta-analysis, generative AI

## Abstract

Social robots are increasingly being integrated into school and home settings to support children’s development. However, their effectiveness in fostering children’s social and emotional learning (SEL) remains insufficiently understood. To address this gap, this study presents a meta-analysis of 66 empirical studies comprising 166 effect sizes and 3468 participants. The findings indicate that social robot interventions produce an overall small-to-moderate positive effect on children’s social and emotional competence. Moderator analyses identified robot role as the only statistically significant moderator of intervention effectiveness. None of the other examined moderator variables showed statistically significant between-group differences. Nevertheless, some descriptive patterns were observed in the estimated effects, with relatively larger effect sizes for cartoon-like and physical robots, individual interaction methods, and generative AI-enabled robots, as well as for longer interventions. Among child characteristics, relatively larger effect sizes were observed for older children, children with special development needs, and those from developed countries. However, these numerical differences should be interpreted cautiously, as they were not supported by statistically significant between-group tests. They therefore do not provide sufficient evidence that these characteristics systematically moderate intervention effectiveness, but may indicate patterns worthy of further investigation. Overall, this study synthesizes the growing evidence base on social robot interventions and provides a basis for identifying potentially relevant intervention characteristics for future research.

## 1. Introduction

Social and emotional learning (SEL) refers to the process through which children acquire a set of social, emotional, and behavioral competencies that include self-awareness, self-management, social awareness, relationship skills, and responsible decision-making (CASEL, 2019). Through the development of these competencies, children are able to build healthy identities, regulate their emotions effectively, set and achieve goals, demonstrate prosocial behaviors, and address social problems. These knowledge and skills not only support children’s healthy development but are also closely associated with academic achievement (Barry et al., 2026; Blewitt et al., 2021).

Current SEL programming has been widely implemented across diverse populations, ranging from typically developing children (e.g., Hagelskamp et al., 2013; Schonert-Reichl et al., 2015) to children with special needs (e.g., Kasari et al., 2012; Locke et al., 2010), and across multiple contexts from classroom-based instruction (e.g., Abry et al., 2017; Rimm-Kaufman et al., 2014) to whole-school ecological systems (e.g., Frazier & Doyle Fosco, 2024; Lee, 2025), with the aim of ensuring that all children benefit from such interventions. Despite growing recognition of its importance, SEL continues to receive insufficient attention and institutional support in mainstream educational settings due to the strong emphasis on academic achievement, limited implementation resources, and inadequate teacher preparation in social and emotional competencies (Durlak et al., 2011; Hemmeter et al., 2008; Reinke et al., 2025).

Nevertheless, recent advances in educational technologies and pedagogical approaches have made the integration of robots into education an important direction for educational innovation (Al Hakim et al., 2022; Belpaeme et al., 2018). As robotic technologies continue to evolve, social attributes have increasingly been embedded into robots, giving rise to social robots. By demonstrating human-like social norms and emotional responsiveness, social robots can interact with humans through speech, gestures, and decision-making (H. Chen et al., 2025; Deublein et al., 2018). These capabilities enable social robots to effectively foster students’ social and emotional competencies (Barry et al., 2026; W. Xie & Lu, 2025).

Social robot interventions aimed at fostering children’s SEL skills and competencies have attracted increasing research attention. Most studies have compared robot-assisted interventions with human-led instruction or other technology-based approaches, but their findings remain inconsistent. Several studies have reported positive effects of social robots on children’s SEL outcomes. For example, Al Hakim et al. (2025) investigated the use of social robots as self-dialogue tools in situated learning within a Japanese hospitality course. Their findings showed that social robots enhanced students’ learning confidence and strengthened their sense of ownership over learning. Similarly, Ros et al. (2016) employed a humanoid robot to promote healthy eating and physical activity among children, reporting higher motivation, greater emotional engagement, and better interaction quality in the robot-assisted group.

In contrast, other studies have found that human-led instruction or alternative technology-based interventions produce more favorable outcomes. For example, Neumann et al. (2023) compared preschool children’s engagement in two learning activities (“Simon Says” and “iPad drawing”) facilitated by either a social robot or a human teacher. Children interacting with the human teacher exhibited significantly higher levels of behavioral and positive emotional engagement. Likewise, Srinivasan et al. (2015) compared rhythm- and robot-based therapies with standard care for children with ASD. Although children initially showed positive engagement with the social robot, their participation declined over time and was accompanied by increased negative behaviors and affective responses.

These mixed findings highlight the need for a comprehensive synthesis of the effectiveness of social robots in promoting children’s SEL. Existing review studies have begun to summarize this growing body of research. However, most are scoping reviews (e.g., Barry et al., 2026; Kewalramani et al., 2024), which primarily provide descriptive overviews of research trends, intervention characteristics, and influencing factors. Although these reviews offer valuable insights into the current state of the field, they cannot quantitatively estimate intervention effects or explain the inconsistencies observed across empirical studies.

To date, only one meta-analysis has quantitatively synthesized this literature. W. Xie and Lu (2025) analyzed 22 empirical studies (38 effect sizes) and reported an overall positive effect of social robot interventions on young children’s SEL skills and competencies. Nevertheless, this study has several limitations. First, its evidence base was relatively small and limited to children aged 0–8 years, restricting the generalizability of its findings. Second, its moderator analysis considered only a limited range of robot design characteristics, providing an incomplete understanding of the conditions under which social robots are most effective.

Building on previous work, the present meta-analysis addresses these limitations by synthesizing a substantially larger body of evidence and extending the target population to children aged 0–18 years. It also provides a more comprehensive examination of factors that may influence intervention effectiveness by considering a variety of moderators across diverse domains: robot characteristics (morphology, robot role, embodiment, interaction type, and the integration of generative AI), contextual factors (intervention duration), and child characteristics (age, children’s developmental status, and region). By identifying both the overall effectiveness of social robot interventions and the conditions under which they are most beneficial, this study aims to provide more robust evidence to inform the design and implementation of future robot-assisted SEL interventions. Accordingly, this meta-analysis addresses the following two research questions:

RQ1: What is the overall effect of social robots on children’s SEL?
RQ2: How do moderator variables modulate the effects of social robots on children’s SEL?

## 2. Methods

The study selection process followed the Preferred Reporting Items for Systematic Reviews and Meta-Analyses (PRISMA) guidelines. The coding procedures, quality assessment, and data analysis strategies employed in this study are described below.

### 2.1. Literature Search

A comprehensive literature retrieval was carried out across multiple major academic databases within a defined time frame (1 January 2002 to 6 February 2026). The selected databases included Web of Science, Scopus, Google Scholar, IEEE, EBSCO, and ACM Digital Library. These sources were chosen to ensure broad coverage of interdisciplinary research relevant to the study topic. The search strategy was developed based on prior studies while considering the current research context. It incorporated several key dimensions, including the target technology, learning domain, participant, and research context (Wang et al., 2025, 2026). The general structure of the search query is presented below:

robot* OR “social robot*” OR “socially assistive robot*” OR “sociable robot*” OR “companion robot*” OR “assistive robot*” AND child* OR adolescent* OR kid* OR infant* OR pupil* OR student* AND “social-emotional” OR “social and emotional” OR SEL OR emotion* AND school* OR preschool OR kindergarten* OR home OR famil*

**Figure 1:**
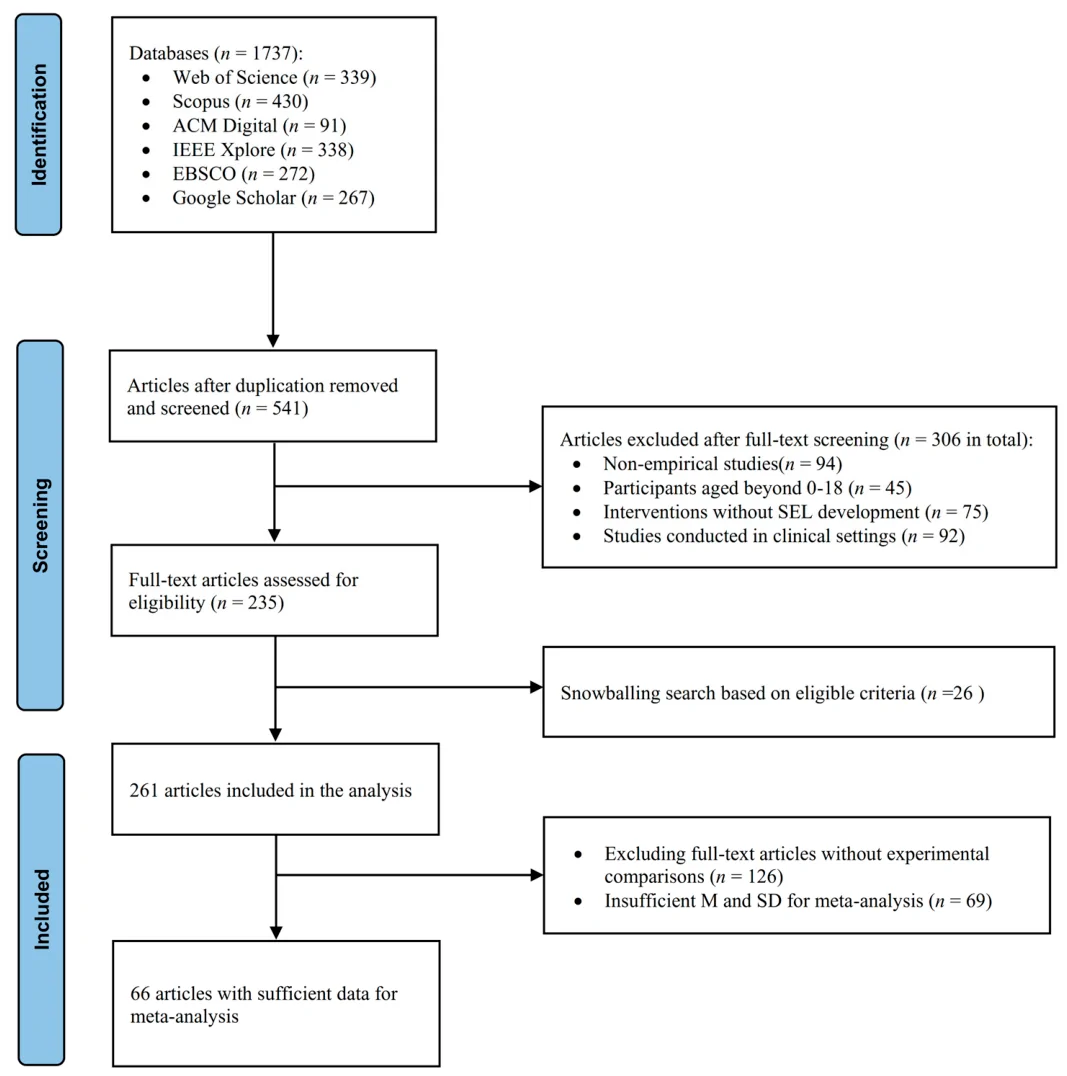
Data search diagram.

To enhance transparency, the overall identification and screening procedure was illustrated using a PRISMA flow diagram in Figure 1.

The multi-round literature search across five electronic databases identified 1737 records, including 339 from Web of Science, 430 from Scopus, 91 from ACM Digital Library, 338 from IEEE Xplore, 272 from EBSCO and 267 from Google Scholar. After merging records retrieved from multiple databases, 1196 duplicate records were removed. The remaining records underwent full-text screening based on the predefined eligibility criteria in Table 1. Subsequently, 235 articles were retained for further assessment. In addition, a snowballing search was conducted by examining the reference lists of relevant review articles and included studies, which identified 26 additional records. After further screening and verification of empirical studies to ensure data validity, 66 studies were ultimately included in the meta-analysis. To ensure reliability, two independent reviewers conducted the screening, and any disagreements were resolved through discussion or consultation with a third reviewer. The characteristics of the included studies, including the number of effect sizes, participants, and other descriptive information, are summarized in Table S1 (see the Supplementary File).

### 2.2. Coding Process and Moderator Variables

To facilitate comparisons across studies and examine potential sources of heterogeneity, a coding framework was developed based on previous studies (Wang et al., 2025, 2026) and the characteristics of the included literature. The framework comprises four categories: (1) robot characteristics (morphology, robot role, embodiment, interaction type, and integration of generative AI); (2) contextual factor (intervention duration); (3) child characteristics (age, children’s development statuses, and region); and (4) SEL skills and competencies.

Following Wang et al. (2025, 2026), robot morphology was classified into three types: anthropomorphic, cartoon-like, and mechanomorphic. Anthropomorphic robots feature human-like forms or characteristics; cartoon-like robots adopt simplified or fictional designs inspired by cartoons, animals, or toys; and mechanomorphic robots exhibit mechanical or industrial appearances. Six robot roles were identified: (a) novice, where robots act as learners or knowledge seekers; (b) peer/partner/companion, where robots engage in reciprocal social interactions; (c) teacher, where robots provide structured instruction and knowledge delivery; (d) teaching assistant/task facilitator, where robots support learning through guidance and prompts; (e) tutor, where robots offer individualized instruction and adaptive feedback; and (f) adaptive, where robots dynamically adjust their roles based on children’s needs. Robot embodiment was categorized as physical or virtual, referring respectively to hardware-based robots and digitally generated agents. Interaction modes were classified as individual (one-to-one interaction) or collective (group-based interaction). Robots were further distinguished as GAI-enabled or conventional, with the former incorporating generative AI models and the latter relying on pre-programmed systems or human control.

Intervention duration was defined as the length of children’s interaction with social robots. Based on the empirical distribution of intervention durations across all included studies, we used a boxplot to identify the first quartile (Q1), median (Q2), and third quartile (Q3), which served as cut-off points for dividing intervention duration into four approximately equal-frequency categories. This approach could help retain sufficient studies in each subgroup without imposing session-based boundaries that were not consistently represented in the literature. Children were categorized into younger and older groups using the grand mean age (8.83 years), considering the wide age range and the uneven distribution of participants across individual age levels. Participants included both typically developing children and children with special needs, such as autism, reading difficulties, or diabetes. Regions were classified according to the United Nations’ World Economic Situation and Prospects (WESP) (United Nations Department of Economic and Social Affairs, 2023) into developed economies, developing economies, and economies in transition.

The coding framework for SEL skills and competencies was grounded in the six-domain framework proposed by Jones et al. (2021), comprising cognitive, emotion, social, values, perspectives, and identity. This framework integrates existing SEL theories, intervention programs, and assessment tools while extending traditional skill-based models to include values, perspectives, and identity. We retained these six domains as the overarching structure and refined the sub-dimensions by integrating the theoretical definitions of the framework with the characteristics and operationalization of SEL-related outcomes observed in the included studies. Thus, the six domains provided the theoretical foundation, while the specific sub-dimensions were informed by the empirical evidence and adapted to the context of social robot-assisted SEL. The final coding framework is presented in Table 2 (see Table S1 for the full details).

The cognitive domain encompasses the core mental processes that support goal-directed behavior, including self-regulation and responsible decision-making, enabling children to effectively navigate academic and interpersonal challenges (Diamond & Lee, 2011). It includes sub-dimensions such as problem-solving (Baker-Henningham et al., 2012; Gaspar et al., 2024) and mental effort (Van Gog et al., 2020).

The emotion domain refers to children’s ability to recognize, express, and regulate their emotions, as well as understand the emotions of others. It includes emotional regulation and empathy and perspective-taking (Jones et al., 2021). The social domain encompasses the interpersonal skills required to build positive relationships, interpret social cues, and engage in effective social interactions. It includes verbal communication, nonverbal communication, and social reciprocity (Prizant et al., 2003).

The values domain concerns the development of prosocial values, habits, and character strengths that promote socially responsible behavior. It includes ethical values, civic values (Jones et al., 2021), and moral development (Burroughs & Barkauskas, 2017). The perspectives domain reflects how children perceive and interpret themselves, others, and their surrounding environment. It includes positive affect, characterized by emotions such as happiness and optimism, and negative affect, characterized by emotions such as sadness, fear, and anxiety. Finally, the identity domain concerns children’s understanding of themselves and their capabilities. It includes self-efficacy (Yüksel et al., 2019), agency (Paterson-Young et al., 2024), and self-awareness (Bahrami et al., 2024; Dubey, 2024).

### 2.3. Effect Size and Testing Heterogeneity

Given the relatively small sample sizes of most included studies, Hedges’ g, a small-sample bias-corrected version of Cohen’s d, was used to estimate standardized effect sizes (Hedges & Olkin, 2014), with values of 0.2, 0.5, and 0.8 indicating small, medium, and large effects, respectively (Cohen, 2013). We calculated Hedges’ g from the means, standard deviations, and sample sizes reported in the original studies. For studies that did not directly report means and standard deviations, we extracted data using WebPlotDigitizer (version 4.8) or estimated them from standard errors and confidence intervals where available (Wang et al., 2025). We used a random-effects model to pool effect sizes because we anticipated considerable between-study variability. Between-study variance (τ^2^) was estimated using the DerSimonian–Laird method, and studies were weighted using the inverse-variance weighting method. We performed all statistical analyses using Stata version 17.

Before effect size calculation, data were preprocessed to ensure appropriate aggregation. To reduce potential dependence among effect sizes, effect sizes were extracted and processed according to participant samples rather than simply by the number of reported outcomes. When a study contained multiple independent samples with no participant overlap, each sample contributed a separate effect size (Weng et al., 2023). When multiple outcomes were reported from the same participant sample without an overall summary measure, these outcomes were combined into a single composite effect size (Cao et al., 2020; Roesler et al., 2021). When several outcomes assessed the same overarching construct, only the most comprehensive outcome was retained (Weng et al., 2023). These procedures were used to minimize dependence among effect sizes before statistical analysis. Subgroup data were also combined when necessary (Pu et al., 2019), such as merging different age groups reported in Di Dio et al. (2020) into a single analytical sample.

Heterogeneity among studies was assessed using Cochran’s Q test and the *I*^2^ statistic. A significant Q value indicates the presence of heterogeneity (Cochran, 1954), while *I*^2^ estimates its magnitude, with values below 25%, 25–75%, and above 75% representing low, moderate, and high heterogeneity, respectively (Higgins & Thompson, 2002).

### 2.4. Sensitivity Analysis and Publication Bias

**Figure 2:**
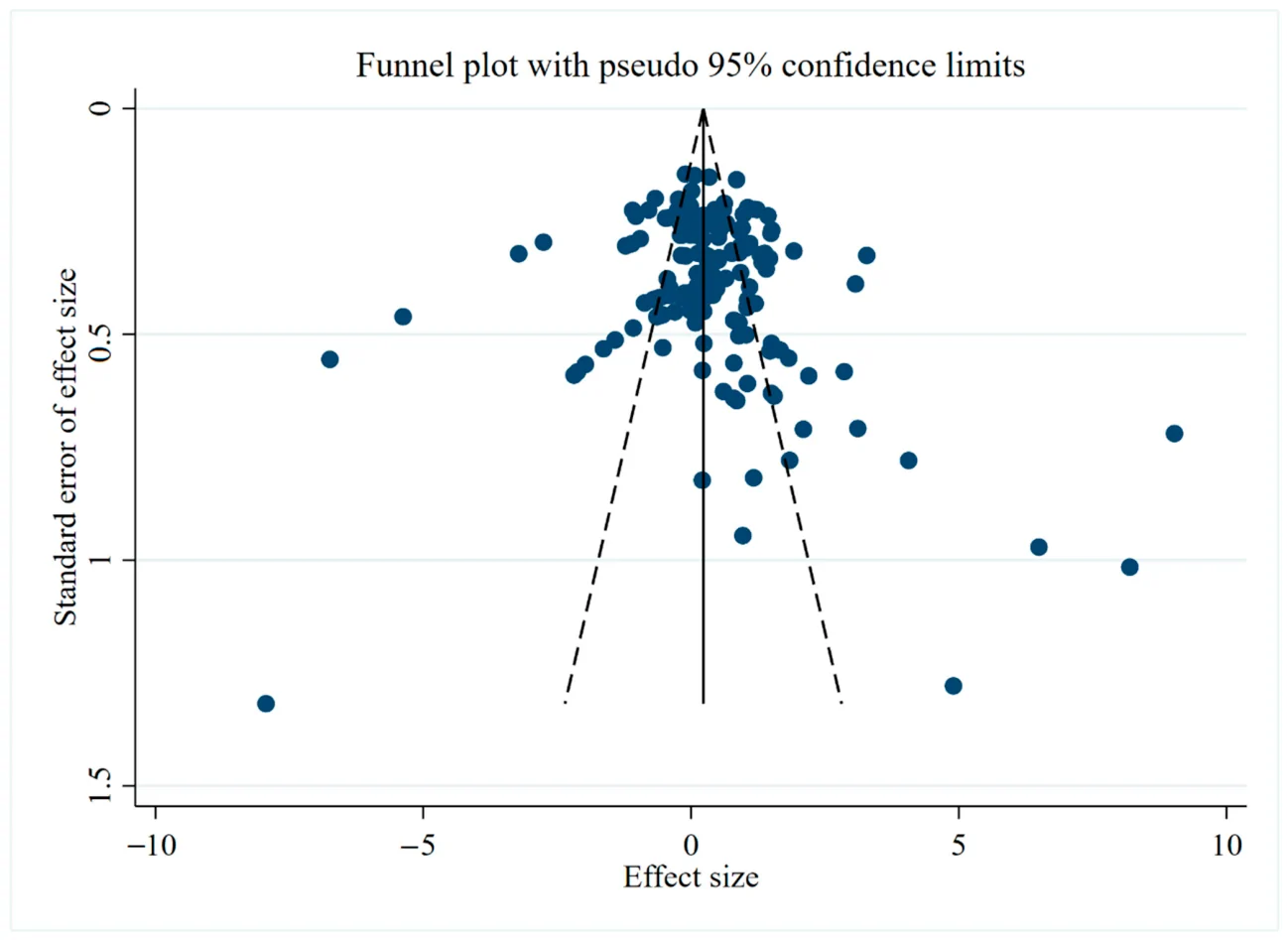
The overall funnel plot.

Publication bias refers to systematic distortion in the research literature when published evidence does not accurately represent the true effect, often because studies with positive or significant findings are more likely to be published. This bias may inflate meta-analytic estimates and affect the accuracy of effect sizes (Franco et al., 2014). In this study, potential publication bias was assessed using a funnel plot and Egger’s test. The funnel plot evaluates the relationship between effect size magnitude (horizontal axis) and standard error (vertical axis), with asymmetry indicating potential bias (Sterne & Harbord, 2004). As shown in Figure 2, the funnel plot showed no pronounced or systematic asymmetry, suggesting no clear evidence of substantial publication bias. Given that visual inspection may be insufficient, particularly with small sample sizes (Sterne et al., 2000), Egger’s test was further conducted to statistically assess funnel-plot asymmetry. The result was non-significant (*p* = 0.156), indicating no statistically significant evidence of funnel-plot asymmetry or substantial publication bias (Egger & Smith, 1998). Nevertheless, the possibility of some degree of publication bias cannot be completely excluded.

**Figure 3:**
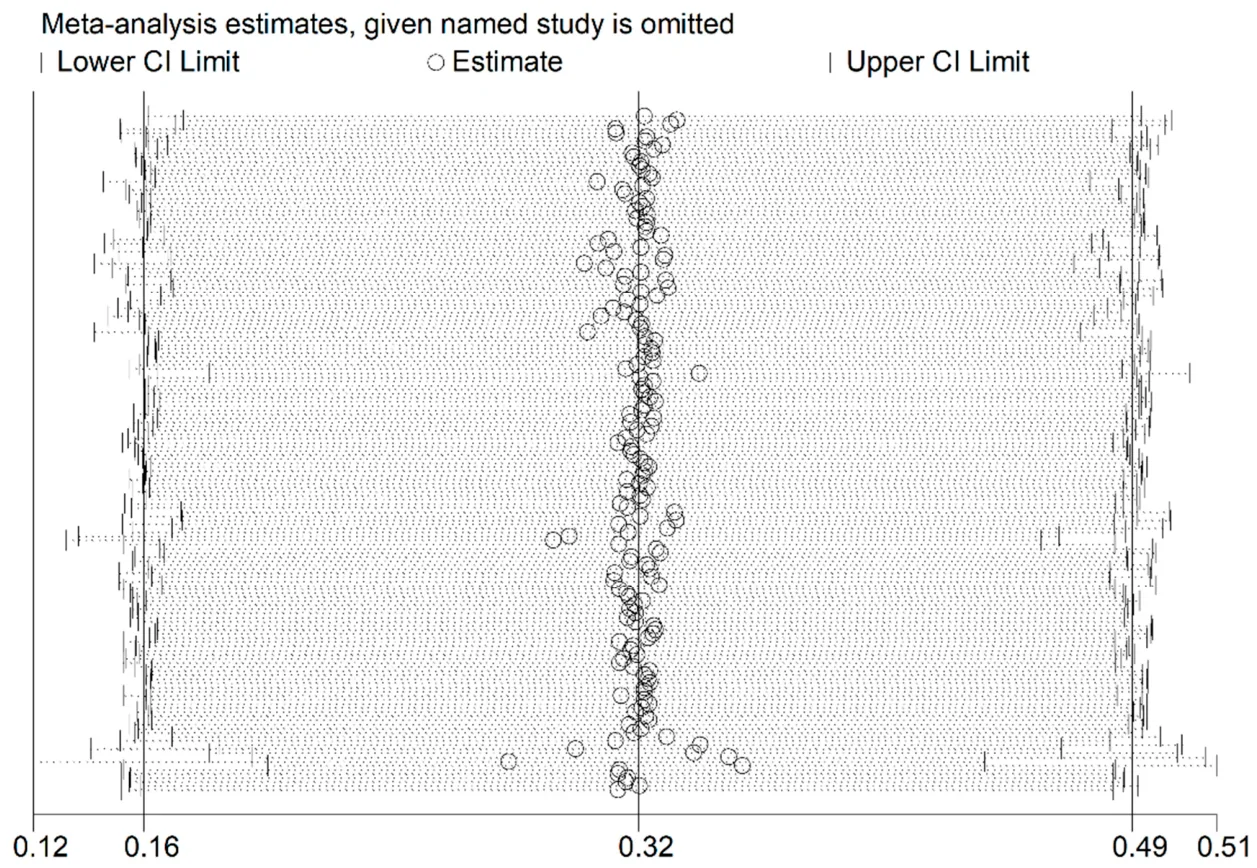
The sensitivity analysis plot.

The potential influence of outliers was also examined, as extreme effect sizes may distort meta-analytic results (Jeong et al., 2019). A leave-one-out analysis was performed by sequentially removing each study and recalculating the pooled effect size (Sahebkar, 2014). As shown in Figure 3, after sequentially removing each study, the pooled effect estimate did not differ significantly from the original overall estimate, indicating no influential outliers. These results confirm the robustness and stability of the meta-analytic findings.

### 2.5. Quality Assessment

Quality assessment is a critical component of meta-analysis (Khan et al., 2001). In this study, the Cochrane Collaboration Risk of Bias Tool was used to evaluate the methodological quality of the included studies (Higgins et al., 2011). The tool assesses six domains: (a) random sequence generation (selection bias), evaluating whether participants were randomly assigned; (b) allocation concealment (selection bias), assessing whether group assignment was adequately concealed; (c) blinding of participants and personnel (performance bias), examining whether awareness of intervention conditions could influence outcomes; (d) blinding of outcome assessment (detection bias), evaluating whether outcome assessors remained independent; (e) incomplete outcome data (attrition bias), assessing the handling and reporting of missing data; and (f) selective reporting (reporting bias), examining whether outcomes were selectively reported. Each domain was rated as having a low, high, or unclear risk of bias.

We adapted the risk-of-bias assessment criteria to reflect the methodological characteristics of educational research. Specifically, for allocation concealment, we considered studies to have a low risk of selection bias when participants had similar baseline characteristics. When information relevant to allocation or group comparability was insufficiently reported, the risk of bias was classified as unclear (Dietrichson et al., 2021).

Regarding blinding of participants and personnel, blinding participants to the research design is almost impossible in educational settings because participants need to know the learning tasks they engage in to facilitate meaningful learning (Ødegaard et al., 2021). Therefore, the included studies in this meta-analysis were generally considered to have an unclear risk of performance bias when participants blinding was not reported.

Similarly, blinding of outcome assessment can be difficult in educational research because outcome assessments frequently rely on children’s self-completed questionnaires, meaning that participants are typically aware of the intervention they received when reporting their experiences and outcomes. Accordingly, the risk of detection bias was evaluated with consideration of the type of outcome measure and the procedures used for outcome assessment. Studies using predetermined or standardized assessment tools, such as validated questionnaires or multiple-choice measures, and/or reporting appropriate procedures for outcome assessment were considered to have a lower risk of detection bias, whereas insufficiently reported assessment procedures resulted in an unclear risk of bias (Kyaw et al., 2019).

## 3. Results

In this section, we report the results of quality assessment, followed by overall effect size of social robot-assisted interventions on children’s SEL skills and competences development, as well as the effect sizes across different moderator variables.

### 3.1. Quality Assessment Results

**Figure 4:**
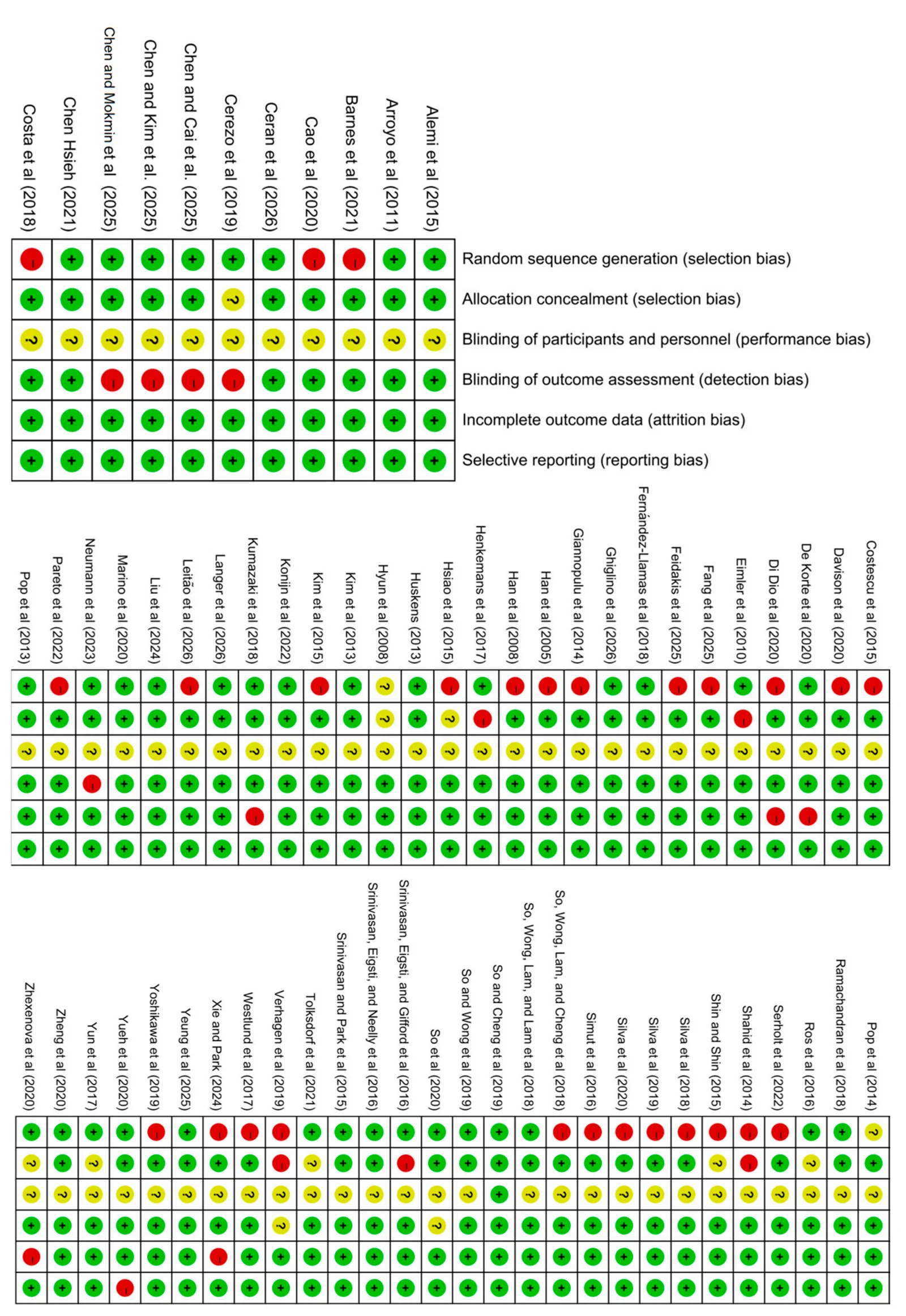
Risk of bias of the included studies. *Note.* (1) Green plus signs (+) indicate low risk of bias, yellow question marks (?) indicate unclear risk of bias, and red minus signs (−) indicate high risk of bias. (2) The following are the citations mentioned in Figure 4: Alemi et al. (2015); Arroyo et al. (2011); Barnes et al. (2021); Cao et al. (2020); Ceran et al. (2026); Cerezo et al. (2019); Chen Hsieh (2021); H. Chen et al. (2025); J. Chen et al. (2025); L. Chen et al. (2025); Costa et al. (2018); Costescu et al. (2015); Davison et al. (2020); De Korte et al. (2020); Di Dio et al. (2020); Eimler et al. (2010); Fang et al. (2025); Feidakis et al. (2025); Fernández-Llamas et al. (2018); Ghiglino et al. (2026); Giannopulu et al. (2014); J. Han et al. (2005); J. H. Han et al. (2008); Henkemans et al. (2017); Hsiao et al. (2015); Huskens et al. (2013); Hyun et al. (2008); E. S. Kim et al. (2013, 2015); Konijn et al. (2022); Kumazaki et al. (2018); Langer et al. (2026); Leitão et al. (2026); Liu et al. (2024); Marino et al. (2020); Neumann et al. (2023); Pareto et al. (2022); Pop et al. (2013, 2014); Ramachandran et al. (2018); Ros et al. (2016); Serholt et al. (2022); Shahid et al. (2014); Shin and Shin (2015); Silva et al. (2018, 2019, 2020); Simut et al. (2016); So et al. (2020, 2019a, 2018a, 2019b, 2018b); Srinivasan et al. (2015, 2016a, 2016b); Tolksdorf et al. (2021); Verhagen et al. (2019); Westlund et al. (2017); B. Xie and Park (2024); Yeung et al. (2025); Yoshikawa et al. (2019); Yueh et al. (2020); Yun et al. (2017); Zheng et al. (2020); Zhexenova et al. (2020).

**Figure 5:**
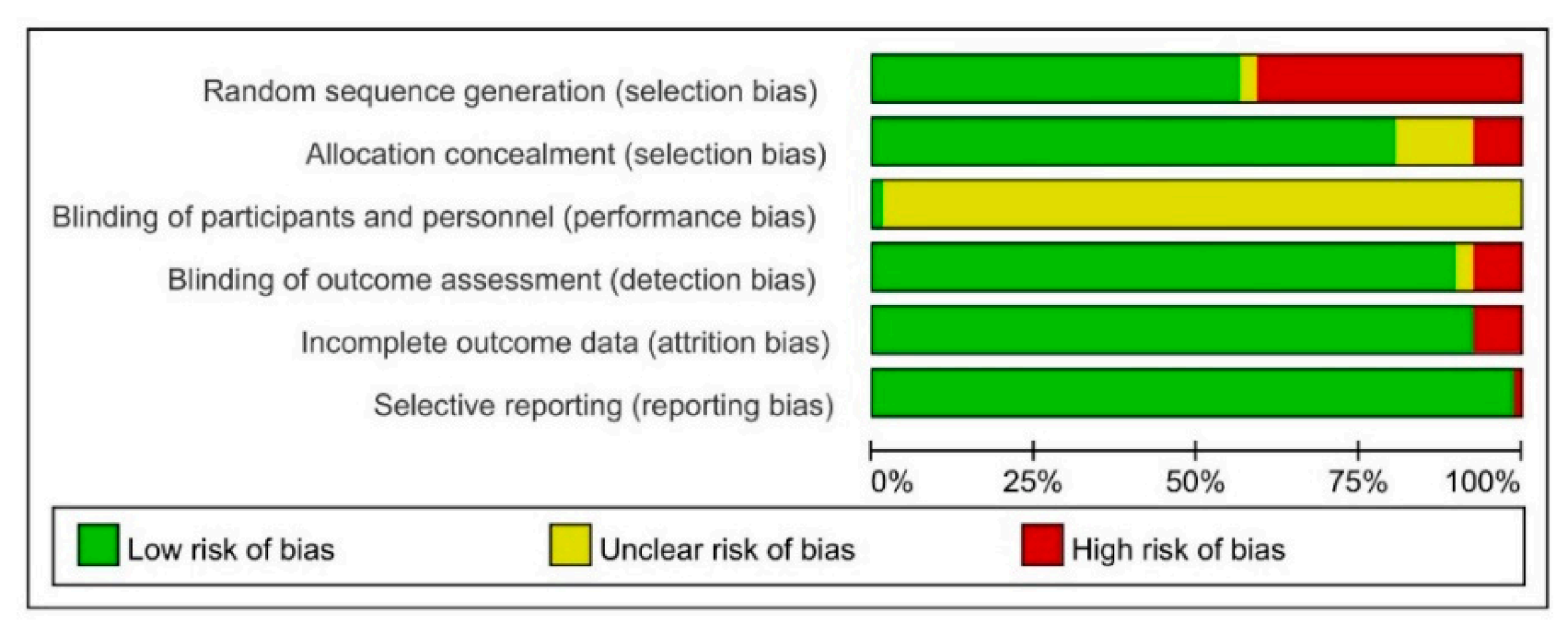
A summary of risk of bias.

Figure 4 presents the risk of bias assessment for each included study across the six domains, while Figure 5 summarizes the overall distribution of risk ratings. Most studies were rated as having a low risk of bias in random sequence generation, allocation concealment, incomplete outcome data, and selective reporting. However, some studies showed a high risk of bias in random sequence generation (*N* = 27), allocation concealment (*N* = 5), and blinding of outcome assessment (*N* = 5). Blinding of participants and personnel was primarily rated as unclear risk, likely because blinding is challenging to implement in educational interventions, where intervention conditions are often apparent to both learners and instructors (Hutchison & Styles, 2010). Overall, most included studies demonstrated low risk across the assessed domains, suggesting generally acceptable methodological quality.

### 3.2. Overall Effect Size

**Figure 6:**
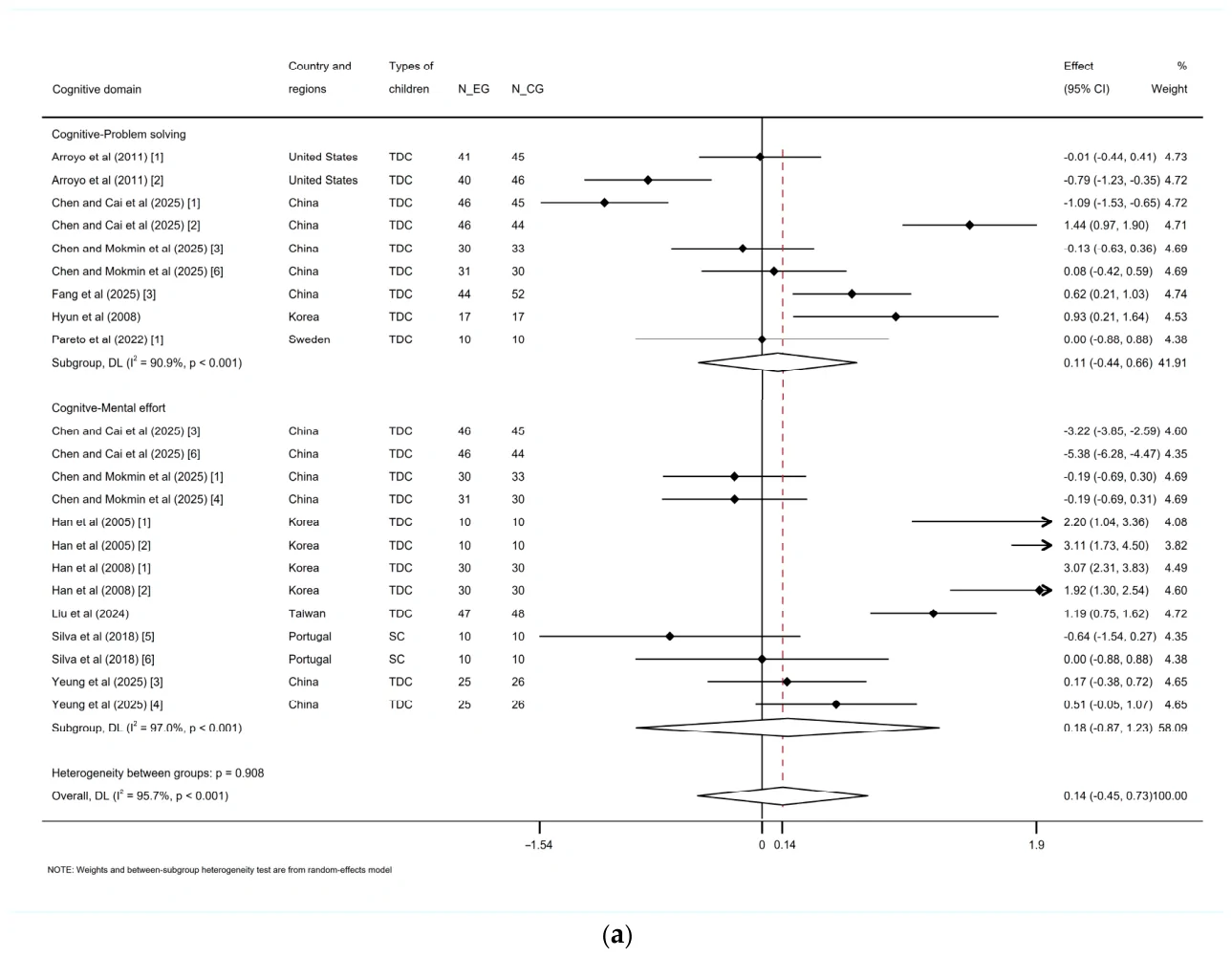

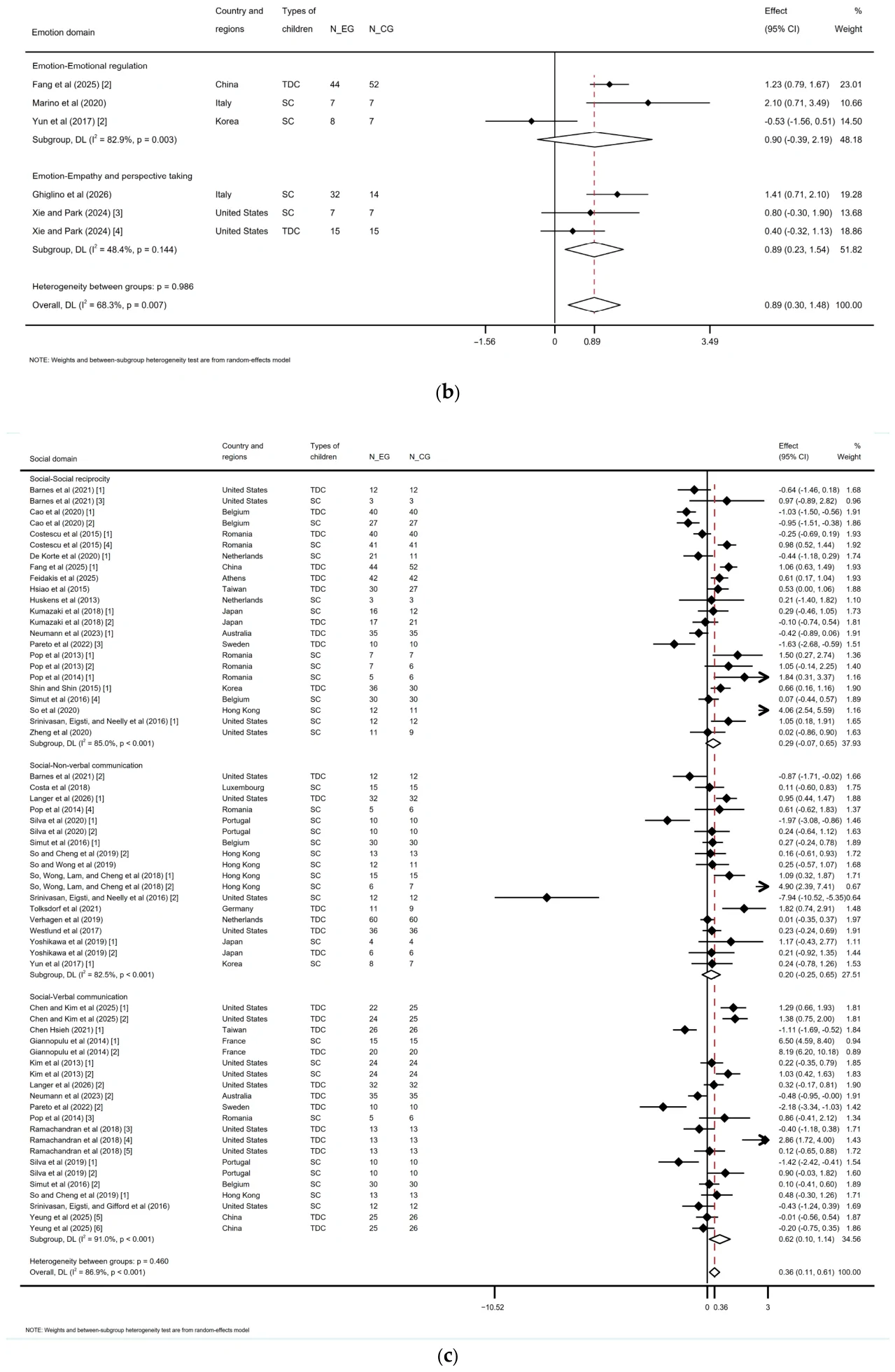

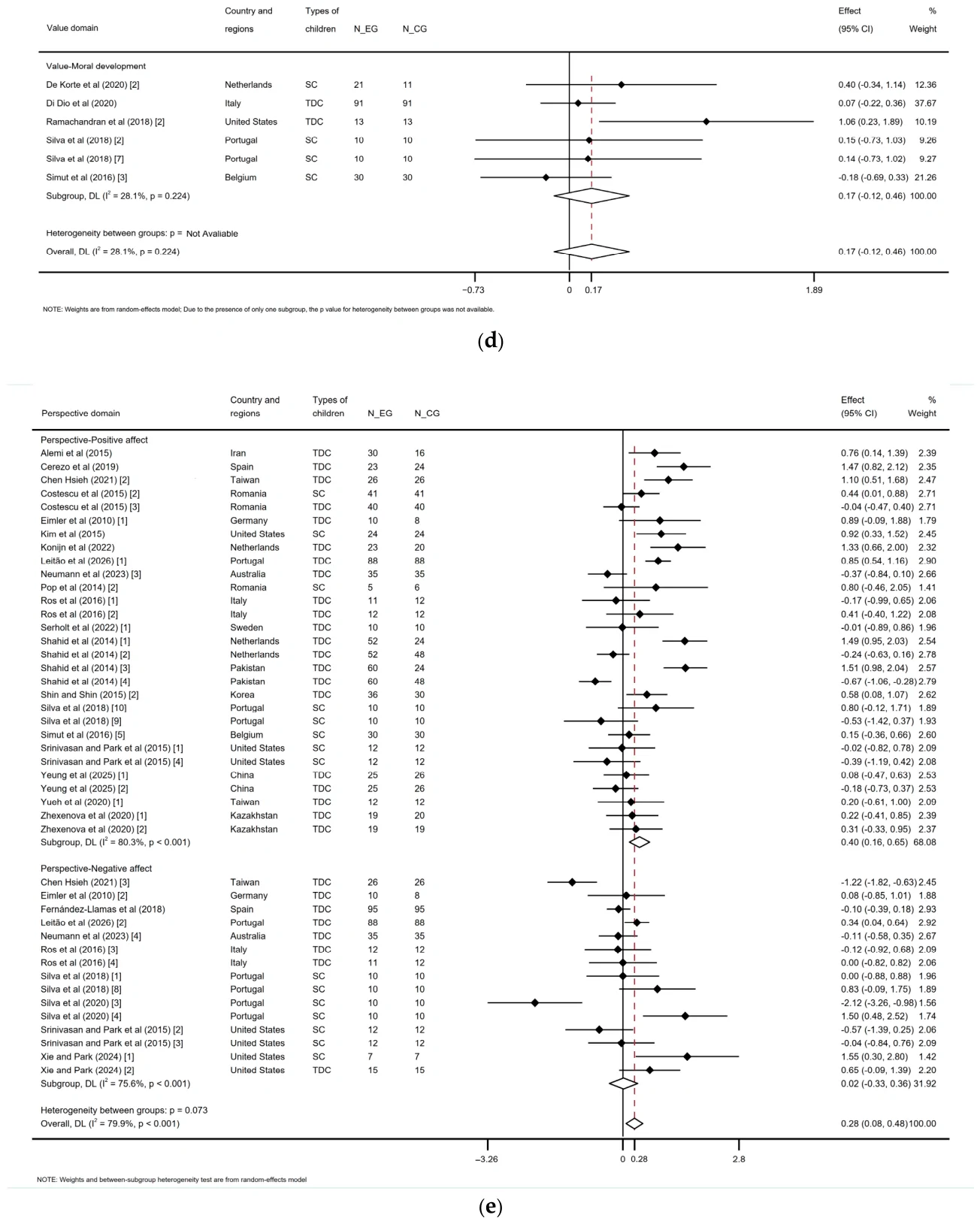

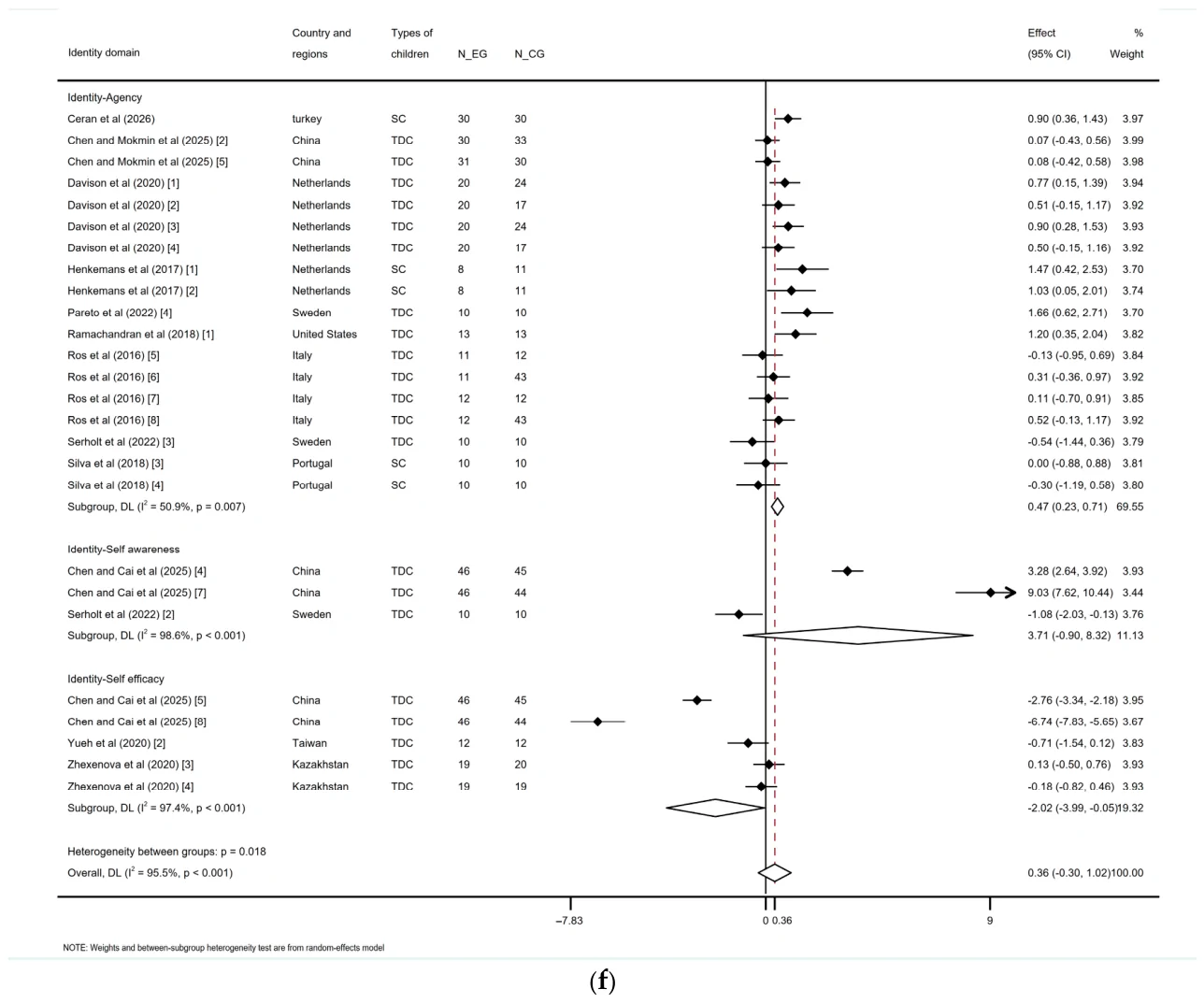
Forest plot of effect sizes for social robots’ impact on children’s SEL competencies and skills across cognitive, emotion, social, value, perspective, and identity domains. (**a**) Forest plot of the cognitive dimension. (**b**) Forest plot of the emotion dimension. (**c**) Forest plot of the social dimension. (**d**) Forest plot of the value dimension. (**e**) Forest plot of the perspective dimension. (**f**) Forest plot of the identity dimension. *Note.* (1) Black diamonds represent the effect estimates of individual studies, while open diamonds represent pooled effect estimates for the corresponding subgroups and the overall analysis. (2) The bracketed numbers (e.g., [1]) following a citation indicate different effect sizes reported in the same study and are used solely to distinguish multiple effect sizes from the same article.

The meta-analysis involving 66 empirical studies with 166 independent effect sizes yielded a significant, small-to-moderate overall positive influence on children’s SEL skills and competences [*k* = 166, Hedges’ *g* = 0.322, 95% confidence interval (CI) [0.157, 0.486], *p* < 0.0001, df = 165, *I*^2^ = 90.70]. We observed substantial heterogeneity (*I*^2^ = 90.70), prompting further exploration of potential sources through subgroup analyses (Song et al., 2001). The result of the overall effect size is shown in Figure 6.

Regarding the specific subdimensions, the intervention demonstrated a strong effect on emotion domain (*k* = 6, Hedges’ *g* = 0.887, 95% CI [0.298, 1.475]). Significant positive results were also observed within the social domain (*k* = 62, Hedges’ *g* = 0.360, 95% CI [0.110, 0.610]) and perspectives domain (*k* = 44, Hedges’ *g* = 0.278, 95% CI [0.076, 0.480]). In contrast, the impacts on cognitive (*k* = 22, Hedges’ *g* = 0.143, 95% CI [−0.477, 0.732]), identity (*k* = 26, Hedges’ *g* = 0.360, 95% CI [−0.302, 1.022]) and values domains (*k* = 6, Hedges’ *g* = 0.172, 95% CI [−0.119, 0.462]) were statistically negligible.

The intervention was effective for both typically developing children (*k* = 102, Hedges’ *g* = 0.280, 95% CI [0.064, 0.497]) and those with special needs (*k* = 64, Hedges’ *g* = 0.385, 95% CI [0.159, 0.611]), with the latter group showing a slightly higher effect, although the difference did not reach statistical significance, *p* = 0.512 > 0.05.

### 3.3. Moderator Analysis

To further investigate the observed heterogeneity, moderator analyses were performed across the conditions outlined in Section 2.2, with findings organized according to robot characteristics, context factors, and child demographics. Table 3 present the results of the moderator analyses for overall learning outcomes, as well as the cognitive, emotional, social, value, perspective, and identity domains.

#### 3.3.1. Robot Characteristics (Morphologies): Numerically Larger Effects for Cartoon-like than Anthropomorphic Robots

As shown in Table 3, anthropomorphic robots were used more widely than cartoon-like robots. The aggregated findings indicate that anthropomorphic robots with human-like features produced a small yet statistically significant improvement in students’ overall SEL skills and competences (*k* = 118, Hedges’ *g* = 0.247, 95% CI [0.039, 0.454]). This effect size was mostly contributed by the perspectives domain (*k* = 26, Hedges’ *g* = 0.251, 95% CI [0.010, 0.491]; see Table S4).

In comparison, robots with cartoon-like appearances demonstrated a moderate-to-large overall effect (*k* = 47, Hedges’ *g* = 0.505, 95% CI [0.244, 0.767]), which was mainly contributed by the social dimension (*k* = 21, Hedges’ *g* = 0.855, 95% CI [0.384, 1.326]; see Table S5). However, no statistically significant difference was found between cartoon-like robots and anthropomorphic robots, *p* = 0.129 > 0.05.

#### 3.3.2. Robot Characteristics (Robot Roles): Adaptive Roles Tend to Yield Strongest Outcomes, Teacher and Novice Roles Show Limited Effects

The effectiveness of social robots varied across instructional roles. Although based on only three studies, robots adopting adaptive roles demonstrated the largest overall effect (*k* = 3, Hedges’ *g* = 1.048, 95% CI [0.522, 1.574]). This effect was entirely driven by improvements in the social domain (Table S5). However, this estimate should be interpreted cautiously given the limited evidence base and potential instability associated with the small number of effect sizes.

Following adaptive roles, robots acting as teaching assistants/task facilitators also yielded a significant and large overall effect (*k* = 6, Hedges’ *g* = 0.834, 95% CI [0.609, 1.058]). These gains were mainly reflected in the social (*k* = 2, Hedges’ *g* = 0.880, 95% CI [0.491, 1.269]; Table S5) and perspectives domains (*k* = 2, Hedges’ *g* = 0.650, 95% CI [0.261, 1.038]; Table S4).

When robots functioned as tutors, they produced a significant moderate overall effect on children’s SEL skills and competencies (*k* = 62, Hedges’ *g* = 0.435, 95% CI [0.227, 0.644]). The strongest improvements were observed in the cognitive domain (*k* = 9, Hedges’ *g* = 1.111, 95% CI [0.320, 1.903]; Table S2), followed by the values (*k* = 2, Hedges’ *g* = 0.701, 95% CI [0.060, 1.342]) (Table S7) and identity domains (*k* = 12, Hedges’ *g* = 0.468, 95% CI [0.247, 0.690]; Table S6). No significant effects were found in the emotion or social domains. The tutor role also appeared more beneficial for typically developing children (*k* = 40, Hedges’ *g* = 0.520, 95% CI [0.274, 0.765]) than for children with special needs (*k* = 22, Hedges’ *g* = 0.243, 95% CI [−0.169, 0.655]), although this difference was not statistically significant (*p* = 0.258; Table S8).

By contrast, when robots were designed as peers/partners/companions, they produced a small but non-significant overall effect on children’s SEL skills and competencies (*k* = 70, Hedges’ *g* = 0.317, 95% CI [−0.019, 0.654]). Nevertheless, significant improvements were observed in the emotion (*k* = 3, Hedges’ *g* = 0.886, 95% CI [0.229, 1.543]; Table S3), social (*k* = 28, Hedges’ *g* = 0.617, 95% CI [0.179, 1.055]; Table S5), and perspectives domains (*k* = 18, Hedges’ *g* = 0.397, 95% CI [0.009, 0.784]; Table S4). Subgroup analysis further indicated that this role was more effective for children with special needs (*k* = 42, Hedges’ *g* = 0.454, 95% CI [0.180, 0.727]) than for typically developing children (*k* = 28, Hedges’ *g* = 0.066, 95% CI [−0.573, 0.706]; Table S8).

Robots acting as novices also showed no significant overall effect (*k* = 12, Hedges’ *g* = −0.121, 95% CI [−0.657, 0.416]), although a significant negative effect was found in the social domain (*k* = 2, Hedges’ *g* = −1.881, 95% CI [−2.655, −1.106]; Table S5). Similarly, robots assuming the teacher role demonstrated a negligible and non-significant overall effect (*k* = 13, Hedges’ *g* = 0.001, 95% CI [−0.209, 0.211]).

#### 3.3.3. Robot Characteristics (Embodiments): Numerically Higher Effects for Physical than Virtual Robots

The overall impact of physical social robots on children’s SEL skills and competences was moderate, demonstrating a statistically significant improvement (*k* = 158, Hedges’ *g* = 0.329, 95% CI [0.156, 0.503]). The most substantial effect was observed in the emotion domain (*k* = 6, Hedges’ *g* = 0.887, 95% CI [0.298, 1.475]; Table S3). Subgroup analysis revealed that children with special needs showed a moderate positive effect (*k* = 63, Hedges’ *g* = 0.375, 95% CI [0.146, 0.605]), which was higher than that observed for typically developing children (*k* = 95, Hedges’ *g* = 0.294, 95% CI [0.061, 0.528]; Table S8).

In contrast, the overall effect of virtual robots on children’s SEL skills and competences was small and statistically insignificant (*k* = 8, Hedges’ *g* = 0.229, 95% CI [−0.150, 0.607]). The comparison between the two groups showed no significant difference, with *p* = 0.636 > 0.05.

#### 3.3.4. Robot Characteristics (Interaction Types): Numerically Higher Effects for Individual than Collective Interaction

The effectiveness of social robots did not differ significantly between collective and individual interaction modes (*p* = 0.731). Under collective interaction, the overall effect on children’s SEL skills and competencies was small and non-significant (*k* = 26, Hedges’ *g* = 0.214, 95% CI [−0.419, 0.846]). Nevertheless, significant improvements were observed in the emotion (*k* = 2, Hedges’ *g* = 1.402, 95% CI [0.724, 2.080]; Table S3) and social domains (*k* = 8, Hedges’ *g* = 0.564, 95% CI [0.082, 1.045]; Table S5). Subgroup analysis showed a significant positive effect for children with special needs (*k* = 2, Hedges’ *g* = 1.318, 95% CI [0.193, 2.442]), although this result was based on only two studies. No significant effect was found for typically developing children (*k* = 24, Hedges’ *g* = 0.117, 95% CI [−0.550, 0.784]; Table S8).

Under individual interaction, social robots produced a significant moderate overall effect on children’s SEL skills and competencies (*k* = 140, Hedges’ *g* = 0.327, 95% CI [0.186, 0.468]). The largest improvement was observed in the cognitive domain (*k* = 17, Hedges’ *g* = 0.591, 95% CI [0.123, 1.058]; Table S2), followed by significant effects in the social (*k* = 54, Hedges’ *g* = 0.332, 95% CI [0.048, 0.617]; Table S5), identity (*k* = 21, Hedges’ *g* = 0.286, 95% CI [0.035, 0.537]; Table S6), and perspectives domains (*k* = 38, Hedges’ *g* = 0.268, 95% CI [0.040, 0.495]; Table S4). Positive effects were comparable for typically developing children (*k* = 78, Hedges’ *g* = 0.310, 95% CI [0.129, 0.491]) and children with special needs (*k* = 62, Hedges’ *g* = 0.354, 95% CI [0.124, 0.583]), with no significant between-group difference (*p* = 0.771; Table S8).

#### 3.3.5. Robot Characteristics (Involvement of Generative Artificial Intelligence (GAI)): Numerically Larger Effects for GAI-Enabled than Conventional Robots

Robots integrated with generative AI (GAI) demonstrated a moderate and statistically significant effect on children’s SEL skills and competencies (*k* = 13, Hedges’ *g* = 0.474, 95% CI [0.127, 0.822]). The largest improvements were observed in the social domains (*k* = 2, Hedges’ *g* = 1.334, 95% CI [0.888, 1.781]; Table S5) and perspectives domains (*k* = 2, Hedges’ *g* = 0.954, 95% CI [0.117, 1.791]; Table S4). Similarly, conventional robots also produced a significant moderate effect, although with a smaller overall effect size (*k* = 153, Hedges’ *g* = 0.307, 95% CI [0.130, 0.484]). Their strongest effects were found in the emotion domains (*k* = 4, Hedges’ *g* = 1.036, 95% CI [0.218, 1.853]; Table S3), social domains (*k* = 60, Hedges’ *g* = 0.322, 95% CI [0.070, 0.574]; Table S5), and perspectives domains (*k* = 42, Hedges’ *g* = 0.251, 95% CI [0.045, 0.457]; Table S4). However, the overall difference between GAI-enabled and conventional robots was not statistically significant (*p* = 0.401).

Subgroup analyses by developmental status revealed positive effects for both robot types. Among typically developing children, GAI-enabled robots (*k* = 11, Hedges’ *g* = 0.403, 95% CI [0.038, 0.768]) and conventional robots (*k* = 91, Hedges’ *g* = 0.267, 95% CI [0.027, 0.506]) showed comparable effects (*p* = 0.540; Table S8). Among children with special neeeds, larger effect sizes were observed for both GAI-enabled robots (*k* = 2, Hedges’ *g* = 1.131, 95% CI [0.303, 1.958]) and conventional robots (*k* = 62, Hedges’ *g* = 0.363, 95% CI [0.134, 0.592]), although the between-group difference remained non-significant (*p* = 0.080; Table S8).

#### 3.3.6. Context Factors (Intervention Duration): Variation in Effect Estimates Across Intervention Durations

Intervention effectiveness varied across session durations. Short sessions (2.45–13.125 min) produced a significant small-to-moderate effect on children’s SEL skills and competencies (*k* = 30, Hedges’ *g* = 0.394, 95% CI [0.079, 0.709]). Moderate sessions (13.125–40 min) showed a comparable overall effect (*k* = 33, Hedges’ *g* = 0.350, 95% CI [0.018, 0.682]), with particularly strong gains in the cognitive domain (*k* = 5, Hedges’ *g* = 2.027, 95% CI [0.925, 3.130]; Table S2).

In contrast, longer sessions (40–90 min) showed no significant overall effect (*k* = 27, Hedges’ *g* = −0.050, 95% CI [−0.712, 0.612]), although a significant improvement was observed in the social domain (*k* = 3, Hedges’ *g* = 0.638, 95% CI [0.281, 0.995]; Table S5). Very long sessions (>90 min) yielded the strongest overall effect (*k* = 28, Hedges’ *g* = 0.682, 95% CI [0.415, 0.949]), indicating a rebound in intervention effectiveness. These interventions were particularly beneficial for the emotion (*k* = 4, Hedges’ *g* = 1.036, 95% CI [0.218, 1.853]; Table S3) and social domains (*k* = 13, Hedges’ *g* = 0.991, 95% CI [0.524, 1.459]; Table S5).

Overall, the effect estimates exhibited a numerical increase–decline–rebound pattern across session durations. However, the overall between-group test indicated that the four session-duration categories did not differ statistically significantly in their effect estimates (*p* = 0.138). Pairwise comparisons showed a significant difference only between the 40–90 min and >90 min groups (*p* = 0.044), whereas all other comparisons were non-significant.

#### 3.3.7. Child Demographics (Age): Numerically Larger Effects for Older than Younger Children

To examine age-related differences, participants were divided into two groups based on the grand mean age of the sample (M = 8.83 years): a younger group (2.5–8.83 years) and an older group (8.83–18.52 years). This data-driven approach ensured a balanced age classification. The overall effects did not differ significantly between the two age groups (*p* = 0.782).

For the younger group, social robots produced a small-to-moderate positive effect on children’s SEL skills and competencies (*k* = 98, Hedges’ *g* = 0.296, 95% CI [0.142, 0.451]). The effect was slightly larger for typically developing children (*k* = 47, Hedges’ *g* = 0.328, 95% CI [0.134, 0.523]) than for children with special needs (*k* = 51, Hedges’ *g* = 0.260, 95% CI [0.000, 0.520]; Table S8).

For the older group, social robots yielded a moderate overall effect (*k* = 68, Hedges’ *g* = 0.350, 95% CI [0.006, 0.693]). Within this group, the effect was smaller and non-significant for typically developing children (*k* = 55, Hedges’ *g* = 0.210, 95% CI [−0.188, 0.607]) but substantially larger for children with special needs (*k* = 13, Hedges’ *g* = 0.831, 95% CI [0.485, 1.177]; Table S8).

#### 3.3.8. Child Demographics (Regions of Residence): Numerically Larger Effects for Developing than Developed Economies

Regional analysis showed that social robot interventions produced a significant positive effect in developed countries (*k* = 114, Hedges’ *g* = 0.301, 95% CI [0.152, 0.449]), whereas the effects were not statistically significant in developing economies (*k* = 48, Hedges’ *g* = 0.390, 95% CI [−0.039, 0.818]) or economies in transition (*k* = 4, Hedges’ *g* = 0.122, 95% CI [−0.195, 0.439]).

Domain-specific analyses further revealed regional differences. In developed countries, significant improvements were observed in the emotion (*k* = 4, Hedges’ *g* = 1.075, 95% CI [0.400, 1.751]; Table S3), identity (*k* = 17, Hedges’ *g* = 0.465, 95% CI [0.171, 0.759]; Table S6), and perspectives domains (*k* = 33, Hedges’ *g* = 0.293, 95% CI [0.073, 0.512]; Table S4). In developing countries, a significant effect was found only in the social domain (*k* = 13, Hedges’ *g* = 0.610, 95% CI [0.112, 1.108]; Table S5), whereas no significant domain-specific effects were identified in economies in transition.

Subgroup analyses by CC showed a similar pattern. Economies in transition included only typically developing children. In both developed and developing countries, interventions generally yielded larger effects for children with special needs (developed: *k* = 56, Hedges’ *g* = 0.317, 95% CI [0.083, 0.551]; developing: *k* = 8, Hedges’ *g* = 0.997, 95% CI [0.159, 1.835]) than for typically developing children (developed: *k* = 58, Hedges’ *g* = 0.286, 95% CI [0.092, 0.481]; developing: *k* = 40, Hedges’ *g* = 0.262, 95% CI [−0.212, 0.736]; Table S8).

## 4. Discussion

This study synthesized findings from 66 studies, involving a total of 3468 participants, and produced 166 effect sizes. The subsequent discussion elaborates the key findings in relation to each research question.

### 4.1. RQ1: What Is the Overall Effect of Social Robots on Children’s SEL?

Our meta-analysis revealed a small-to-moderate overall positive effect of social robot interventions (Hedges’ *g* = 0.322), indicating that social robots can effectively support improvements in children’s SEL skills and competencies. Nevertheless, the substantial heterogeneity across studies indicates that the true intervention effects vary considerably. Consequently, the pooled effect size should be viewed as an average estimate across diverse contexts rather than a universally applicable effect. This finding highlights the importance of considering contextual and methodological factors when interpreting intervention outcomes and underscores the need to identify the conditions under which social robot interventions are more effective.

Our finding yielded a smaller effect size than W. Xie and Lu (2025), who found a larger effect of early social robot-based interventions on children’s social-emotional improvement (Hedges’ *g* = 0.651). Several factors may explain this difference. First, W. Xie and Lu (2025) focused exclusively on children aged 0–8 years, whereas our study included a broader age range (0–18 years), which may introduce greater developmental variability. Second, our study adopted the six-domain SEL framework proposed by Jones et al. (2021), which captures a broader range of SEL dimensions than previous studies. The broad framework may reduce the overall effect size because it expands the range of measured outcomes to include domains that may be less sensitive to social robot interventions. Third, while W. Xie and Lu (2025) focused specifically on SEL programs, our meta-analysis included a broader range of educational contexts, where SEL-related outcomes were sometimes indirectly assessed through behaviors such as turn-taking, cooperation, and trust toward robots (Barry et al., 2026), rather than standardized SEL measures.

For comparison, F. Zhang et al. (2023) reported a similar magnitude of effect for virtual reality-based interventions targeting children’s SEL (Hedges’ *g* = 0.378). Although differences in intervention characteristics and study designs should be considered, the broadly similar effect sizes observed across these two technology modalities provide a preliminary indication that both VR and social robot-assisted interventions may have potential for supporting children’s SEL improvement. More importantly, despite differences in technological forms (e.g., virtual reality and social robots), comparable effect sizes indicate that intervention effectiveness may depend less on the technology itself and more on how interventions are designed and implemented (Cipriano et al., 2023). Therefore, further investigation of specific intervention features and contextual moderators is needed to better understand the conditions under which social robot interventions are most effective.

Among the six SEL domains, the emotion domain demonstrated the largest effect, followed by the social domain, both showing significant improvements. These findings may be explained by the socially interactive and embodied characteristics of robots. Through modalities such as gestures, posture, and gaze, social robots can enhance perceived social presence and encourage children to engage in interaction and communication (Belpaeme et al., 2018; Deng et al., 2019; Y. Kim & Tscholl, 2021). Moreover, supportive functions, such as guided dialogue and adaptive feedback, can increase children’s engagement, reduce learning-related anxiety, and promote confidence (Triantafyllidis et al., 2023). Thus, sustained interaction and social scaffolding may represent key mechanisms underlying the positive effects of social robots on children’s social and emotional improvement.

### 4.2. RQ2: How Do Moderator Variables Modulate the Effects of Social Robots on Children’s SEL?

The moderator analyses indicated that robot roles were the only moderator showing a statistically significant difference, whereas the comparisons across the other examined moderators were not statistically significant. Therefore, interpretations of the non-significant moderator findings should be considered exploratory and descriptive. The summarized results are as follows:

**Robot characteristics:**

(1) Morphology: Robots with a cartoon-like appearance showed numerically larger effects than anthropomorphic robots.
(2) Robot roles: Robots taking on adaptive roles demonstrated greater effectiveness than those acting in other roles.
(3) Embodiment: Physical robots showed numerically larger effects than virtual robots.
(4) Interaction type: Individual interaction showed a numerically larger effect than collective interaction.
(5) Integration of GAI: Social robots integrated with GAI showed numerically larger effects than conventional robots in fostering children’s SEL development.

**Demographic factors:**

(1) Age: Older children (8.83 to 18.52 years) showed numerically larger effects than younger children (2.5 to 8.83 years).
(2) Regions: Studies conducted in developing countries showed numerically larger effects.

**Contextual factors** (Intervention duration): Longer interventions (>90 min) exhibited numerically larger effects than shorter interventions.

The subgroup analyses provide further insight into how robot design influences the effectiveness of social robot interventions for promoting children’s SEL skills and competencies. Regarding robot morphology, cartoon-like robots yielded numerically larger effect sizes than humanoid robots. However, the between-group difference was not statistically significant. This descriptive pattern may be partly related to the potential limitations associated with highly anthropomorphic designs. Specifically, highly anthropomorphic robots may evoke the uncanny valley effect when their appearance exceeds their social and emotional capabilities, leading to discomfort and increased cognitive load during interaction (Mori et al., 2012). Such reactions may reduce children’s willingness to communicate and engage emotionally, thereby limiting opportunities for developing social and emotional competencies. In contrast, children generally perceive cartoon-like robots as more approachable and emotionally safe (J. Zhang et al., 2020), which may encourage emotional expression, social interaction, and cooperative behaviors. This interpretation is consistent with Henschel et al. (2021), who suggested that children’s acceptance and comfort are more critical than anthropomorphism itself in shaping interaction outcomes. Moreover, cartoon-like robots are often integrated with playful interaction and positive emotional expressions, which have been shown to reduce anxiety, increase social engagement, and foster emotional well-being (Lin et al., 2014; Rudenko et al., 2024). From an ethical perspective, less anthropomorphic robots may also reduce the risk of children confusing robots with humans, thereby supporting the improvement of appropriate social understanding (Boada et al., 2021; Torras, 2024).

Regarding robot roles, social robots adopting adaptive roles produced the largest effect sizes, although this finding was based on a relatively small number of studies. The limited evidence base may reduce the stability and reliability of this finding. However, adaptive robot roles remain a promising design feature that warrants further investigation in future research. The potential benefit of adaptive roles lies in their flexibility, which enables robots to dynamically switch between instructional, collaborative, and social-emotional functions according to children’s needs, thereby creating richer opportunities to develop communication, emotional regulation, cooperation, and social problem-solving skills (Feidakis et al., 2025). This interpretation is consistent with Rohlfing et al. (2022), who argued that adaptive role switching supports more natural and sustainable human–robot interactions while addressing children’s evolving learning and emotional needs. Teaching assistant/task facilitator was the second most effective role. Unlike teacher-centered approaches, these robots primarily provide scaffolding, prompts, and feedback that encourage children to actively participate, collaborate with peers, and regulate their own learning and emotions (Papakostas et al., 2021; Woo et al., 2021). Such supportive interactions are well aligned with the goals of SEL, as they foster autonomy, cooperation, and social responsibility rather than passive knowledge acquisition. Consistent with Wang et al. (2025), teaching assistant/task facilitator roles facilitate individualized scaffolding and human–robot collaboration, enabling children to practice social and emotional competencies in authentic learning contexts. Although tutors also demonstrated positive effects, their impact was smaller, suggesting that facilitative and adaptive roles may be more effective than purely instructional roles for promoting children’s SEL.

As for robot embodiment, physical robots showed numerically larger effect estimates than virtual robots, although the between-group difference was not statistically significant. This finding should also be interpreted cautiously because the relatively small number of studies involving virtual robots may have limited the statistical power to detect an effect. One possible explanation for this finding is that physical robots provide a stronger sense of social presence and embodied interaction, making it easier for children to establish emotional connections and engage in reciprocal communication (Gasteiger et al., 2021). Such embodied interactions are particularly relevant to SEL, as they encourage emotional expression, empathy, cooperation, and other interpersonal skills through face-to-face engagement (Belpaeme et al., 2018; Severson et al., 2024). In contrast, virtual robots lack physical presence and tangible interaction, which may limit opportunities for developing social connectedness and emotional engagement (Oliveira et al., 2021). This interpretation is supported by Wang et al. (2026), who found that embodied robots are more likely to foster trust and emotional bonding, both of which are fundamental to children’s SEL improvement.

Concerning interaction type, one-to-one interactions produced larger overall effects than collective interactions. Individual interactions allow robots to provide personalized emotional support, adaptive scaffolding, and immediate feedback, thereby better addressing children’s individual social and emotional needs (Belpaeme et al., 2018). Nevertheless, collective interactions demonstrated notable benefits in the emotion and social domains, suggesting that group-based activities offer valuable opportunities to practice cooperation, communication, and peer interaction. Rather than viewing the two interaction types as competing approaches, they may support different aspects of SEL. Individual interactions may be more effective for developing intrapersonal competencies, whereas collective interactions may better foster interpersonal competencies. Future studies should therefore investigate how different interaction formats can be integrated to maximize SEL outcomes.

Regarding the integration of GAI, GAI-enabled robots produced numerically larger effects than conventional robots, although the between-group difference was not statistically significant. Moreover, this finding was based on a relatively small number of GAI-related effect sizes, which may limit the stability and generalizability of the observed pattern and therefore warrant cautious interpretation. The observed descriptive pattern may be consistent with emerging evidence suggesting that GAI has the potential to support children’s SEL through more personalized and responsive interactions (Chigora, 2024; Henriksen et al., 2025) and largely aligns with the meta-analysis of Feng et al. (2025). From an SEL perspective, GAI enables robots to engage in more natural conversations, recognize children’s emotional states, and provide context-sensitive feedback and social scaffolding (Gaba et al., 2026; Verhelst et al., 2025). These capabilities may enable robots to better support emotional regulation, perspective taking, and social communication by adapting interactions to children’s developmental needs. As social robot interventions increasingly emphasize personalized and emotionally responsive support, integrating GAI may represent a promising direction for future SEL interventions. However, given the limited number of studies and effect sizes involving GAI-enabled robots, together with the non-significant between-group difference, further research is needed to determine whether GAI provides additional benefits over conventional social robots.

The second group of moderators concerns demographic factors. Regarding children’s age, our subgroup analyses showed numerically larger effect sizes among older children than younger children, although the between-group difference was not statistically significant. This numerical pattern may reflect that older children face more complex social and emotional demands, requiring advanced competencies in emotion regulation, perspective-taking, communication, and social problem-solving. Social robots can provide structured interaction, guided feedback, and repeated opportunities for practicing these skills, thereby better supporting older children’s SEL improvement (Muir et al., 2023; W. Xie & Lu, 2025). In addition, older children may possess greater cognitive capacity to engage in meaningful interactions with robots and benefit from robot-mediated scaffolding (Wang et al., 2026). Overall, these findings suggest that social robots may be particularly valuable for supporting SEL improvement as children encounter increasingly complex social environments.

As for regions, the economies in transition subgroup, represented by only four effect sizes, showed a non-significant intervention effect. Although significant intervention effects were observed in developed countries but not in developing countries, the between-group difference was not statistically significant. This disparity may reflect differences in the maturity of SEL implementation. Developed countries have generally established more comprehensive SEL curricula, teacher preparation, and educational resources, enabling social robots to complement existing practices more effectively (OECD, 2015; Yuan et al., 2026). In addition, studies from developed countries included a larger proportion of children with special needs, who benefited more from social robot interventions in the present meta-analysis, which may have further contributed to the regional differences (Blewitt et al., 2021; Wigelsworth et al., 2022).

Among the contextual factors, longer interventions were associated with numerically larger effect sizes in the present study. This numerical pattern is consistent with previous meta-analyses reporting significantly higher effects for longer interventions in other settings (Donnermann et al., 2025). However, intervention duration did not significantly moderate intervention effectiveness in the present study. One possible explanation is that, unlike academic skills, SEL develops through repeated social interaction and emotional experiences. Interestingly, intervention effects followed a non-linear pattern, declining initially before increasing again. The initial decline may reflect the fading novelty effect in human–robot interaction (Reimann et al., 2024; Smedegaard, 2022). As children become familiar with the robot, interactions shift from novelty-driven engagement to more authentic social exchanges, allowing the benefits for SEL to emerge more fully over time (Lambert et al., 2020).

## 5. Contribution

This study makes three key contributions to the fields of social robotics and SEL, with the primary contribution being a comprehensive understanding of the effectiveness, mechanisms, and boundary conditions of social robot interventions for children’s SEL development.

First, this meta-analysis provides the most comprehensive quantitative synthesis to date of the effects of social robots on children’s SEL skills and competencies. By integrating evidence from a substantially larger number of studies and participants than previous reviews, this study offers a more robust estimate of intervention effects across diverse contexts. These findings strengthen the empirical foundation of social robot-assisted SEL and provide evidence-based guidance for future intervention development.

Second, this study advances the conceptualization of SEL in technology-mediated learning environments. Given the substantial heterogeneity in existing social robot research, particularly in the measurement and operationalization of SEL outcomes, this study develops a multidimensional SEL framework by synthesizing established SEL theories and evidence from 66 empirical studies. This framework integrates socio-emotional, cognitive, and belief-related dimensions, providing a more comprehensive lens for understanding how SEL develops through human–robot interaction.

Third, this study identifies when and under what conditions social robots are most effective through systematic moderator analyses. By examining factors related to robot design, intervention characteristics, and child demographics, this study reveals the critical role of design configurations and contextual conditions in shaping intervention outcomes. These findings move beyond evaluating whether social robots work and provide practical insights into how to design more adaptive, effective, and inclusive SEL interventions.

## 6. Implications

This study provides several implications for the application of social robots in supporting children’s SEL development. First, educational institutions and practitioners may consider integrating social robots into structured and pedagogically grounded SEL activities rather than treating them as standalone technological tools. The effectiveness of social robots depends not only on their presence but also on how they are embedded within learning scenarios targeting specific SEL competencies. With advances in interactive capabilities and the integration of GAI, social robots are expected to provide increasingly adaptive and personalized support for children’s SEL development (Papadakis & Lampropoulos, 2026).

Second, designers and practitioners are advised to prioritize children’s psychological comfort, autonomy, and ethical protection when developing and implementing social robots. The findings suggest that robot characteristics, such as morphology and role, influence intervention effectiveness. Therefore, designs should emphasize child-friendly appearances, adaptive interactions, and opportunities for children to exercise agency during activities. At the same time, anthropomorphic features should be carefully calibrated to avoid misleading children about robots’ social capabilities. Researchers and practitioners should ensure informed assent, transparent communication, and appropriate safeguards for privacy and data protection (Boada et al., 2021; Torras, 2024). Given current technological limitations, social robots should be viewed as supportive partners rather than replacements for human educators, with appropriate human supervision remaining essential (LeTendre & Gray, 2024).

Third, future interventions are suggested to adopt longer and developmentally appropriate designs to capture the sustained effects of social robots on SEL. Although short-term interactions may benefit from novelty effects, extended interventions are more likely to reveal stable improvements by allowing children to develop familiarity and meaningful interaction patterns with robots. However, considering children’s limited tolerance for prolonged engagement, interventions should be structured into multiple phases to maintain participation while enabling longitudinal evaluation. Furthermore, the stronger effects observed among older children highlight the importance of designing age-sensitive interventions that address increasingly complex social and emotional challenges across development.

## 7. Limitations and Future Work

Although this study provides valuable insights into the role of social robots in supporting children’s SEL development, several limitations warrant consideration. First, regarding the inclusion criteria, this study incorporated both journal articles and conference proceedings to capture a comprehensive range of evidence on social robot-based SEL interventions. However, conference papers may involve less rigorous peer-review processes and often present preliminary findings, which may contribute to greater heterogeneity and influence the robustness of the estimated effects.

Second, regarding the analytical approach, the data synthesis method adopted in this study followed established procedures commonly used in previous meta-analytic research. Although this approach is widely accepted, more advanced methods, such as three-level meta-analytic models, could further enhance the precision of effect size estimation by explicitly accounting for dependencies among multiple effect sizes extracted from the same studies.

Third, the evidence base for GAI-integrated social robots remains limited due to the emerging nature of this research area. Although our findings provide preliminary evidence regarding the potential contribution of GAI-enabled social robots to children’s SEL development, future research with a larger number of empirical studies may enable more comprehensive analyses of additional moderators and further clarify the conditions under which social robots effectively support SEL.

Fourth, some moderator categories were represented by relatively few effect sizes, for instance, adaptive robot roles and GAI-enabled robots. This limitation primarily reflects the limited number of empirical studies examining these conditions. The small number of effect sizes reduces the precision of the corresponding estimates and makes the findings more sensitive to variation across individual studies. Thus, these subgroup findings should be interpreted cautiously rather than as definitive evidence of differential intervention effects. Future empirical research examining these moderators is needed to provide a more robust evidence base and clarify whether these observed patterns are replicable.

Fifth, the developed versus developing economy classification reflects broad country-level economic contexts and may not capture substantial within-country variation in educational resources, school conditions, and socioeconomic circumstances. Thus, this moderator should be interpreted as a broad contextual indicator at the country level rather than as a proxy for the conditions of individual children or schools, and its findings should not be generalized to these lower levels.

Sixth, study design and the potential novelty effect of social robots should be considered. Study design was not examined as a moderator or sensitivity variable. Given the practical challenges of implementing true RCTs in school-based educational settings, we did not distinguish between RCTs and quasi-experimental studies, limiting our ability to determine whether study design contributed to differences in the estimated intervention effects. In addition, many of the included interventions were single-session or short-term, and the observed effects may therefore partly reflect children’s initial curiosity or novelty associated with interacting with robots. Future research should include more long-term follow-up studies to examine the sustained effects of social robot interventions on children.

## Figures and Tables

**Table 1:** Inclusion criteria.

Dimension	Criteria
Study type	Peer-reviewed journal articles and conference proceedings.
Research topic	Interventions targeting the effects of social robots on children’s SEL skills and competencies.
Research setting	Social robot interventions implemented in formal (e.g., schools) or informal (e.g., homes) educational settings, rather than clinical settings.
Participant	Participants aged 0–18 years, consistent with the United Nations’ definition of children (UNCRC, 2005).
Research design	(Quasi-)experimental studies comparing social robot interventions with non-social robot control conditions.
Research outcome	Studies reporting SEL-related outcomes or behavioral indicators of SEL (e.g., turn-taking, cooperation, and trust) and sufficient data for effect size calculation (e.g., sample size, means, and standard deviations).

**Table 2:** Children’s SEL skills and competencies framework.

Code	Sub-Dimension
Cognitive domain	Problem solving
	Mental effort
Emotion domain	Emotional regulation
	Empathy and perspective taking
Social domain	Verbal communication
	Non-verbal communication
	Social reciprocity
Value domain	Moral development
Perspective domain	Positive affect
	Negative affect
Identity domain	Self-efficacy
	Agency
	Self-awareness

*Note.* Full information about each dimension and sub-dimension can be accessed from Table S1 in the Supplementary File.

**Table 3:** The moderator analysis for overall outcomes.

Moderator	Category	*k*	*g*	95% CI	*z*	*p*	Q_B_	*df*	*p*
Robot morphology	Anthropomorphic	118	0.247	(0.039, 0.454)	2.330	0.020			
	Cartoon-like	47	0.505	(0.244, 0.767)	3.786	0.000			
	Total between						2.300	1	0.129
Robot roles	Peer/Partner/Companion	70	0.317	(−0.019, 0.654)	1.848	0.065			
	Tutor	62	0.435	(0.227, 0.644)	4.089	0.000			
	Teaching assistant/Task facilitator	6	0.834	(0.609, 1.058)	7.275	0.000			
	Novice	12	−0.121	(−0.657, 0.416)	−0.440	0.660			
	Teacher	13	0.001	(−0.209, 0.211)	0.005	0.996			
	Adaptive roles	3	1.048	(0.522, 1.574)	3.908	0.000			
	Total between						38.010	5	0.000
Robot embodiments	Physical	158	0.329	(0.156, 0.503)	3.716	0.000			
	Virtual	8	0.229	(−0.150, 0.607)	1.182	0.237			
	Total between						0.220	1	0.636
Interaction type	Individual interaction	140	0.327	(0.186, 0.468)	4.546	0.000			
	Collective interaction	26	0.214	(−0.419, 0.846)	0.662	0.508			
	Total between						0.120	1	0.731
Involvement of GAI	Conventional robot	153	0.307	(0.130, 0.484)	3.408	0.001			
	GAI	13	0.474	(0.127, 0.822)	2.674	0.007			
	Total between						0.710	1	0.401
Intervention duration (min)	2.45–13.125	30	0.394	(0.079, 0.710)	2.450	0.014			
	13.125–40	33	0.350	(0.018, 0.682)	2.068	0.039			
	40–90	27	−0.050	(−0.712, 0.612)	−0.148	0.882			
	More than 90	28	0.682	(0.415, 0.949)	4.998	0.000			
	Total between						5.510	3	0.138
Age (years old)	2.5–8.83	98	0.296	(0.142, 0.451)	3.753	0.000			
	8.83–18.52	68	0.350	(0.006, 0.693)	1.994	0.046			
	Total between						0.080	1	0.782
Region classification	Developed economies	114	0.301	(0.152, 0.449)	3.972	0.000			
	Developing economies	48	0.390	(−0.039, 0.818)	1.781	0.075			
	Economies in transition	4	0.122	(−0.195, 0.439)	0.756	0.450			
	Total between						1.280	2	0.528
Children’s development statuses	Typical development children	102	0.280	(0.064, 0.497)	2.536	0.011			
	Special children	64	0.385	(0.159, 0.611)	3.341	0.001			
	Total between						0.430	1	0.512

## Data Availability

The data that support the findings of this study are available from the corresponding author upon reasonable request.

## Supplementary Materials

The following supporting information can be downloaded at: https://www.mdpi.com/article/10.3390/jintelligence14100246/s1, Table S1: Children’s SEL skills and competencies framework, Table S2: The moderator analysis for cognitive domain, Table S3: The moderator analysis for emotion domain, Table S4: The moderator analysis for perspective domain, Table S5: The moderator analysis for social domain, Table S6: The moderator analysis for identity domain, Table S7: The moderator analysis for value domain, Table S8: Moderator effects of social robot interventions on children’s SEL outcomes by developmental status.
