# Supplementary material for: Meta-Analyzing the Impacts of Social Robots on Children’s Social and Emotional Development over Two Decades
Source: J Intell. 2026 Oct 4;14(10):246. doi: 10.3390/jintelligence14100246 (PMC13705870; doi:10.3390/jintelligence14100246)
Supplement: Supplementary file 1 [file jintelligence-14-00246-s001.zip › jintelligence-4534616-supplementary.pdf]

Supplementary File

Table S1. Children's SEL skills and competencies framework

Code	Definition	Sub-dimension	Interpretation
Cognitive domain	The cognitive domain involves basic mental skills for managing behavior and achieving goals, including executive function, self-regulation, decision-making, and problem-solving. These competencies enable children to sustain attention, control impulses, remember and follow instructions, plan and achieve goals, adapt to changing situations, and use information effectively to make decisions and solve problems.	Problem solving	The abilities to devise plans, create strategies, set and achieve goals, and make decisions to solve problems.
		Mental effort	Personal mental engagement that an individual invests when performing a cognitive task. It reflects the intensity of cognitive resource allocation, such as attention, concentration, and deliberate thinking, required to process information or achieve a learning goal.
Emotion domain	The emotion domain includes a set of skills and competencies that help children recognize, express, and control their emotions as well as understand and empathize with others.	Emotional regulation	The capacity to recognize, understand, manage, and respond to emotions in oneself and others, enabling individuals to maintain an optimal emotional state that aligns with social and environmental demands and supports adaptive responses.
		Empathy and perspective taking	The ability to take the perspective of and empathize with others, regardless of their ethnicity, background, or culture.
Social domain	It encompasses the skills to work collaboratively, solve social problems, build positive relationships, and coexist peacefully with others. It also includes the ability to understand and respond to social cues, as well as to interpret and express emotions, feelings, and intentions through verbal and nonverbal communication, such as body language and tone of voice.	Verbal communication	Tone of voice and language enable children to express their needs and emotions, convey messages, and engage in conversations.
		Non-verbal communication	Children interpret and use body language, such as facial expressions, eye contact, and gestures, to communicate feelings in both emotional and interpersonal domains.
		Social reciprocity	Skills for meaningful mutual interaction, including sharing experiences, taking turns, and showing mutual interest.

Value domain	The values domain includes a set of values, skills/competencies, habits, and character strengths that support children to be prosocial and productive members of a particular community.	Moral development	Understanding and acting according to social norms and moral reasoning.
Perspective domain	A child’s perspective is how they view and approach the world. It impacts how they see themselves, others, and their own circumstances as well as influences how they interpret and approach challenges.	Positive affect	A positive outlook on the world, reflected in a learner's emotional state characterized by feelings such as happiness, joy and delight.
		Negative affect	A negative outlook on the world, reflected in a learner's emotional state characterized by feelings such as sad, scared, nervous, angry and anxiety.
Identity domain	Identity encompasses how children understand and perceive themselves and their abilities, such as their knowledge and beliefs about who they are and their ability to learn and grow.	Self-efficacy	Children's understanding and perception of themselves and their abilities, including their knowledge and beliefs about who they are and their capacity to learn and grow.
		Agency	A sense of volition, choice, and self-direction in one’s actions and learning, along with the personal responsibility, psychological investment, and commitment one feels toward their own growth.
		Self-awareness	The capacity to develop and maintain a coherent understanding of oneself over time, including one's personality traits, interests, preferences, strengths, and weaknesses.

Table S2. The moderator analysis for cognitive domain.

Moderator	Category	*k*	*g*	95 % CI	*z*	*p*	Q_B	*df*	*p*
Robot appearance	Anthropomorphic	19	0.134	(-0.522, 0.790)	0.400	0.689			
	Cartoon-like	3	0.235	(-0.940, 1.410)	0.391	0.696			
	Total between						0.020	1	0.883
Robot roles	Peer/Partner/Companion	8	-1.191	(-2.405, 0.022)	-1.924	0.054			
	Tutor	9	1.111	(0.320, 1.903)	2.753	0.006			
	Novice	2	0.657	(-0.500, 1.813)	1.113	0.266			
	Teacher	2	0.341	(-0.051, 0.733)	1.703	0.089			
	Total between						9.980	3	0.019
Robot embodiments	Physical	17	0.146	(-0.646, 0.938)	0.362	0.717			
	Virtual	5	0.159	(-0.397, 0.714)	0.560	0.576			
	Total between						0.000	1	0.980
Interaction type	Individual interaction	17	0.591	(0.123, 1.058)	2.477	0.013			
	Collective interaction	5	-1.497	(-3.470, 0.475)	-1.488	0.137			
	Total between						4.080	1	0.043
Involvement of GAI	Conventional robot	17	0.146	(-0.646, 0.938)	0.362	0.717			
	GAI	5	0.159	(-0.397, 0.714)	0.560	0.576			
	Total between						0.000	1	0.980
Intervention duration (min)	2.45-13.125	2	0.341	(-0.051, 0.733)	1.703	0.089			
	13.125-40	5	2.027	(0.925, 3.130)	3.605	0.000			
	40-90	6	-1.475	(-2.964, 0.013)	-1.943	0.052			
	more than 90	6	0.241	(-0.225, 0.706)	1.013	0.311			
	Total between						14.890	3	0.002
Age (years old)	2.5-8.83	8	0.110	(-0.315, 0.536)	0.508	0.611			
	8.83-18.52	14	0.178	(-0.759, 1.114)	0.372	0.710			
	Total between						0.020	1	0.898
Region classification	Developed economies	5	-0.317	(-0.720, 0.087)	-1.538	0.124			
	Developing economies	17	0.273	(-0.468, 1.015)	0.722	0.470			
	Total between						1.880	1	0.171
Children's development statuses	Typical development children	20	0.188	(-0.441, 0.816)	0.584	0.559			
	Special children	2	-0.309	(-0.939, 0.320)	-0.964	0.335			
	Total between						1.200	1	0.274

Table S3. The moderator analysis for emotion domain.

Moderator	Category	*k*	*g*	95 % CI	*z*	*p*	Q_B	*df*	*p*
Robot appearance	Anthropomorphic	6	0.887	(0.298, 1.475)	2.952	0.003			
	Total between						0.000	0	1.000
Robot roles	Peer/Partner/Companion	3	0.886	(0.229, 1.543)	2.643	0.008			
	Tutor	2	0.743	(-1.827, 3.313)	0.567	0.571			
	Total between						0.010	1	0.916
Robot embodiments	Physical	6	0.887	(0.298, 1.475)	2.952	0.003			
	Total between						0.000	0	1.000
Interaction type	Individual interaction	4	0.564	(-0.226, 1.354)	1.400	0.162			
	Collective interaction	2	1.402	(0.724, 2.080)	4.054	0.000			
	Total between						2.490	1	0.115
Involvement of GAI	Conventional robot	4	1.036	(0.218, 1.835)	2.484	0.013			
	GAI	2	0.522	(-0.084, 1.127)	1.689	0.091			
	Total between						0.980	1	0.322
Intervention duration (min)	more than 90	4	1.036	(0.218, 1.835)	2.484	0.013			
	Total between						0.000	0	1.000
Age (years old)	2.5-8.83	4	1.036	(0.218, 1.835)	2.484	0.013			
	8.83-18.52	2	0.522	(-0.084, 1.127)	1.689	0.091			
	Total between						0.980	1	0.322
Region classification	Developed economies	4	1.075	(0.400, 1.751)	3.121	0.002			
	Developing economies	2	0.418	(-1.299, 2.134)	0.477	0.633			
	Total between						0.490	1	0.485
Children's development statuses	Typical development children	2	0.868	(0.063, 1.673)	2.113	0.035			
	Special children	4	0.915	(-0.113, 1.943)	1.745	0.081			
	Total between						0.000	1	0.944

Table S4. The moderator analysis for perspective domain.

Moderator	Category	*k*	*g*	95 % CI	*z*	*p*	Q_B	*df*	*p*
Robot appearance	Anthropomorphic	26	0.251	(0.010, 0.491)	2.044	0.041			
	Cartoon-like	17	0.352	(-0.049, 0.753)	1.719	0.086			
	Total between						0.180	1	0.671
Robot roles	Peer/Partner/Companion	18	0.397	(0.009, 0.784)	2.006	0.045			
	Tutor	16	0.266	(-0.092, 0.625)	1.456	0.146			
	Teaching assistant/Task facilitator	2	0.650	(0.261, 1.038)	3.274	0.001			
	Novice	3	0.210	(-0.190, 0.609)	1.028	0.304			
	Teacher	5	-0.135	(-0.324, 0.055)	-1.395	0.163			
	Total between						16.880	4	0.002
Robot embodiments	Physical	44	0.278	(0.076, 0.480)	2.697	0.007			
	Total between						0.000	0	1.000
Interaction type	Individual interaction	38	0.268	(0.040, 0.495)	2.309	0.021			
	Collective interaction	6	0.328	(-0.153, 0.810)	1.336	0.182			
	Total between						0.050	1	0.824
Involvement of GAI	Conventional robot	42	0.251	(0.045, 0.457)	2.388	0.017			
	GAI	2	0.954	(0.117, 1.791)	2.233	0.026			
	Total between						2.560	1	0.110
Intervention duration (min)	2.45-13.125	14	0.332	(-0.082, 0.746)	1.571	0.116			
	13.125-40	8	0.233	(-0.284, 0.750)	0.882	0.378			
	40-90	6	0.243	(-0.049, 0.536)	1.632	0.103			
	Total between						0.140	2	0.934
Age (years old)	2.5-8.83	25	0.217	(-0.019, 0.453)	1.801	0.072			
	8.83-18.52	19	0.369	(0.009, 0.729)	2.006	0.045			
	Total between						0.480	1	0.489
Region classification	Developed economies	33	0.293	(0.073, 0.512)	2.608	0.009			
	Developing economies	9	0.237	(-0.345, 0.818)	0.799	0.424			
	Economies in transition	2	0.268	(-0.181, 0.717)	1.171	0.242			
	Total between						0.040	2	0.982
Children's development statuses	Typical development children	29	0.323	(0.066, 0.580)	2.465	0.014			
	Special children	15	0.216	(-0.160, 0.592)	1.124	0.261			
	Total between						0.210	1	0.644

Table S5. The moderator analysis for social domain.

Moderator	Category	*k*	*g*	95 % CI	*z*	*p*	Q_B	*df*	*p*
Robot appearance	Anthropomorphic	41	0.125	(-0.163, 0.412)	0.849	0.396			
	Cartoon-like	21	0.855	(0.384, 1.326)	3.556	0.000			
	Total between						6.720	1.000	0.010
Robot roles	Peer/Partner/Companion	28	0.617	(0.179, 1.055)	2.763	0.006			
	Tutor	21	0.227	(-0.193, 0.648)	1.060	0.289			
	Teaching assistant/Task facilitator	2	0.880	(0.491, 1.269)	4.437	0.000			
	Novice	2	-1.881	(-2.655, -1.106)	-4.758	0.000			
	Teacher	6	0.025	(-0.409, 0.460)	0.114	0.909			
	Adaptive roles	3	1.048	(0.522, 1.574)	3.908	0.000			
	Total between						49.440	5.000	0.000
Robot embodiments	Physical	62	0.360	(0.110, 0.610)	2.827	0.005			
	Total between						0.000	0	1.000
Interaction type	Individual interaction	54	0.332	(0.048, 0.617)	2.287	0.022			
	Collective interaction	8	0.564	(0.082, 1.045)	2.296	0.022			
	Total between						0.660	1.000	0.417
Involvement of GAI	Conventional robot	60	0.322	(0.070, 0.574)	2.503	0.012			
	GAI	2	1.334	(0.888, 1.781)	5.859	0.000			
	Total between						14.970	1.000	0.000
Intervention duration (min)	2.45-13.125	14	0.394	(0.079, 0.710)	1.937	0.053			
	13.125-40	12	0.350	(0.018, 0.682)	-0.598	0.550			
	40-90	3	-0.050	(-0.712, 0.612)	3.502	0.000			
	more than 90	13	0.682	(0.415, 0.949	4.158	0.000			
	Total between						11.970	3.000	0.007
Age (years old)	2.5-8.83	47	0.397	(0.116, 0.679)	2.766	0.006			
	8.83-18.52	15	0.247	(-0.324, 0.819)	0.849	0.396			
	Total between						0.210	1.000	0.645
Region classification	Developed economies	49	0.288	(-0.001, 0.577)	1.955	0.051			
	Developing economies	13	0.610	(0.112, 1.108)	2.399	0.016			
	Total between						1.200	1.000	0.274
Children's development statuses	Typical development children	28	0.246	(-0.101, 0.593)	1.391	0.164			
	Special children	34	0.472	(0.104, 0.841)	2.511	0.012			
	Total between						0.770	1.000	0.381

Table S6. The moderator analysis for identity domain.

Moderator	Category	*k*	*g*	95 % CI	*z*	*p*	Q_B	*df*	*p*
Robot appearance	Anthropomorphic	23	0.383	(-0.363, 1.130)	1.006	0.314			
	Cartoon-like	3	0.264	(-0.522, 1.050)	0.659	0.510			
	Total between						0.050	1	0.829
Robot roles	Peer/Partner/Companion	9	0.459	(-1.817, 2.735)	0.395	0.693			
	Tutor	12	0.468	(0.247, 0.690)	4.141	0.000			
	Novice	5	-0.025	(-0.754, 0.703)	-0.068	0.945			
	Total between						1.610	2	0.446
Robot embodiments	Physical	23	0.368	(-0.427, 1.162)	0.907	0.365			
	Virtual	3	0.340	(-0.184, 0.865)	1.271	0.204			
	Total between						0.000	1	0.955
Interaction type	Individual interaction	21	0.286	(0.035, 0.537)	2.230	0.026			
	Collective interaction	5	0.719	(-2.936, 4.374)	0.386	0.700			
	Total between						0.050	1	0.817
Involvement of GAI	Conventional robot	24	0.389	(-0.361, 1.138)	1.017	0.309			
	GAI	2	0.074	(-0.278, 0.426)	0.412	0.680			
	Total between						0.550	1	0.456
Intervention duration (min)	13.125-40	6	0.180	(-0.538, 0.898)	0.491	0.623			
	40-90	11	0.469	(-1.244, 2.182)	0.537	0.592			
	more than 90	3	0.340	(-0.184, 0.865)	1.271	0.204			
	Total between						0.170	2	0.920
Age (years old)	2.5-8.83	9	0.229	(-0.109, 0.568)	1.329	0.184			
	8.83-18.52	17	0.468	(-0.561, 1.497)	0.892	0.373			
	Total between						0.190	1	0.666
Region classification	Developed economies	17	0.465	(0.171, 0.759)	3.100	0.002			
	Developing economies	7	0.292	(-2.033, 2.616)	0.246	0.806			
	Economies in transition	2	-0.023	(-0.470, 0.425)	-0.100	0.920			
	Total between						3.190	2	0.203
Children's development statuses	Typical development children	21	0.304	(-0.490, 1.098)	0.751	0.453			
	Special children	5	0.606	(0.004, 1.208)	1.972	0.049			
	Total between						0.350	1	0.533

Table S7. The moderator analysis for value domain.

Moderator	Category	*k*	*g*	95 % CI	*z*	*p*	Q_B	*df*	*p*
Robot appearance	Anthropomorphic	3	0.406	(-0.155, 0.967)	1.419	0.156			
	Cartoon-like	3	-0.050	(-0.442, 0.343)	-0.247	0.805			
	Total between						1.700	1	0.192
Robot roles	Peer/Partner/Companion	4	0.026	(-0.208, 0.259)	0.217	0.828			
	Tutor	2	0.701	(0.060, 1.342)	2.143	0.032			
	Total between						3.760	1	0.052
Robot embodiments	Physical	6	0.172	(-0.119, 0.462)	1.157	0.247			
	Total between						0.000	0	1.000
Interaction type	Individual interaction	6	0.172	(-0.119, 0.462)	1.157	0.247			
	Total between						0.000	0	1.000
Involvement of GAI	Conventional robot	6	0.172	(-0.119, 0.462)	1.157	0.247			
	Total between						0.000	0	1.000
Intervention duration (min)	13.125-40	2	0.393	(-0.818, 1.604)	0.636	0.525			
	Total between						0.000	0	1.000
Age (years old)	2.5-8.83	5	0.060	(-0.163, 0.283)	0.529	0.597			
	Total between						0.000	0	1.000
Region classification	Developed economies	6	0.172	(-0.119, 0.462)	1.157	0.247			
	Total between						0.000	0	1.000
Children's development statuses	Typical development children	2	0.483	(-0.475, 1.442)	0.988	0.323			
	Special children	4	0.050	(-0.296, 0.397)	0.284	0.776			
	Total between						0.690	1	0.405

Table S8. Moderator effects of social robot interventions on children's SEL outcomes by developmental status.

		Children's development statuses	
		Typical development children	Special children
Robot appearance	Anthropomorphic	0.194 (-0.047, 0.435)	0.419 (0.031, 0.808)
	Cartoon-like	0.886 (0.314, 1.458)	0.357 (0.081, 0.633)
Robot roles	Peer/Partner/Companion	0.066 (-0.573, 0.706)	0.454 (0.180, 0.727)
	Tutor	0.520 (0.274, 0.765)	0.243 (-0.169, 0.655)
	Teaching assistant/Task facilitator	0.834 (0.609, 1.058)	-
	Novice	-0.121 (-0.657, 0.416)	-
	Teacher	0.001 (-0.209, 0.211)	-
	Adaptive roles	1.048 (0.522, 1.574)	-
Robot embodiments	Physical	0.294 (0.061, 0.528)	0.329 (0.156, 0.503)
	Virtual	0.137 (-0.252, 0.527)	-
Interaction type	Individual interaction	0.310 (0.129, 0.491)	0.354 (0.124, 0.583)
	Collective interaction	0.117 (-0.550, 0.784)	1.318 (0.193, 2.442)
Involvement of GAI	Conventional robot	0.267 (0.027, 0.506)	0.363 (0.134, 0.592)
	GAI	0.403 (0.038, 0.768)	1.131 (0.303, 1.958)
Intervention duration (min)	2.45-13.125	0.349 (0.005, 0.692)	0.522 (-0.282. 1.326)
	13.125-40	0.432 (-0.001, 0.865)	0.079 (-0.139, 0.296)
	40-90	-0.149 (-0.841, 0.542)	1.236 (0.518, 1.954)
	More than 90	0.602 (0.292, 0.912)	0.868 (0.333, 1.404)
Age (years old)	2.5-8.83	0.328 (0.134, 0.523)	0.260 (0.000, 0.520)
	8.83-18.52	0.210 (-0.188, 0.607)	0.831 (0.485, 1.177)
Region classification	Developed economies	0.286 (0.092, 0.481)	0.317 (0.083, 0.551)
	Developing economies	0.262 (-0.212, 0.736)	0.997 (0.159, 1.835)
	Economies in transition	0.122 (-0.195, 0.439)	-
